# A data-based large-scale model for primary visual cortex enables brain-like robust and versatile visual processing

**DOI:** 10.1126/sciadv.abq7592

**Published:** 2022-11-02

**Authors:** Guozhang Chen, Franz Scherr, Wolfgang Maass

**Affiliations:** Institute of Theoretical Computer Science, Graz University of Technology, Graz, Austria.

## Abstract

We analyze visual processing capabilities of a large-scale model for area V1 that arguably provides the most comprehensive accumulation of anatomical and neurophysiological data to date. We find that this brain-like neural network model can reproduce a number of characteristic visual processing capabilities of the brain, in particular the capability to solve diverse visual processing tasks, also on temporally dispersed visual information, with remarkable robustness to noise. This V1 model, whose architecture and neurons markedly differ from those of deep neural networks used in current artificial intelligence (AI), such as convolutional neural networks (CNNs), also reproduces a number of characteristic neural coding properties of the brain, which provides explanations for its superior noise robustness. Because visual processing is substantially more energy efficient in the brain compared with CNNs in AI, such brain-like neural networks are likely to have an impact on future technology: as blueprints for visual processing in more energy-efficient neuromorphic hardware.

## Teaser

A biologically detailed large-scale model for primary visual cortex can robustly solve multiple visual processing tasks.

## INTRODUCTION

The comprehensive model (*1*) for a patch of cortical area V1 in mouse provides an unprecedented window into the dynamics of this brain area. We show that it also provides a unique tool for studying brain-style visual processing and neural coding.

**Fig. 1.**
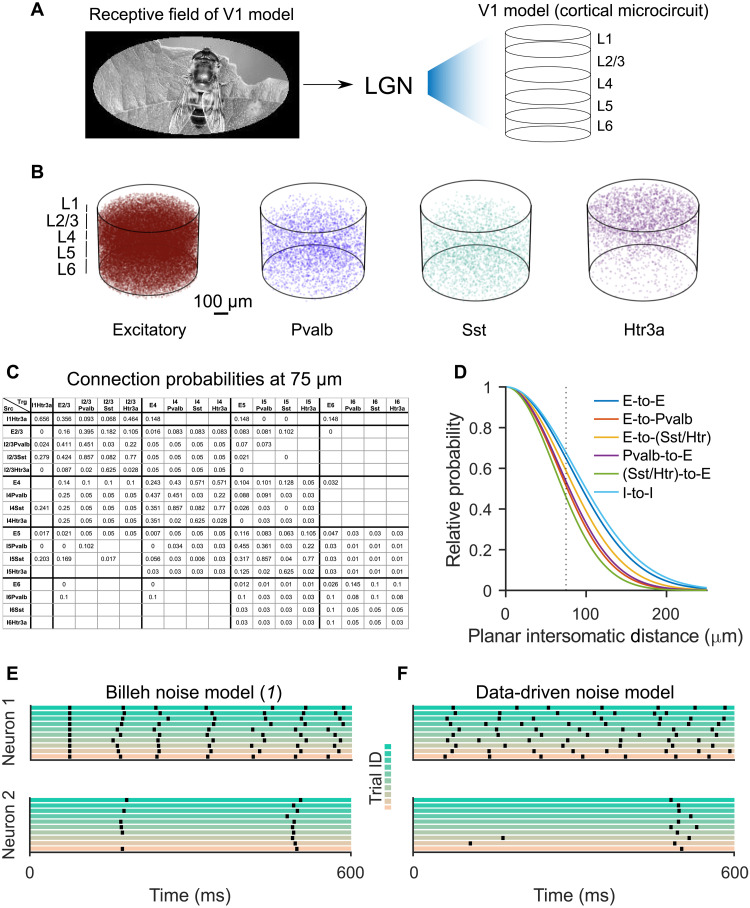
V1 model of (*1*). (**A**) The model consists of four classes of neurons on five layers. It comes together with a model for LGN, which transforms visual inputs into input currents to neurons in the V1 model. The LGN model receives visual input from an oval in the central part of an image (*1*). (**B**) The model contains one excitatory and three inhibitory neuron classes. Each dot denotes the position of a neuron. (**C**) The data-based base connection probabilities of (*1*) depend on the cell class to which the presynaptic (row labels) and postsynaptic neuron (column labels) belongs. White grid cells denote unknown values. (**D**) The base connection probability from (C) is multiplied according to (*1*) for any given pair of neurons by an exponentially decaying factor that depends on the lateral distance between them. Note that the illustrations in (A), (C), and (D) were created by us and derived from the publicly available data provided in (*1*). (**E**) Spike outputs of two randomly selected neurons from the V1 model for 10 trials with the same input (a trial of visual change detection task for natural images), using the noise model of (*1*). (**F**) Same as in (E) but for the version of the data-driven noise model with *s* = *q* = 2 that we used as default-noise model during testing. It causes substantially larger trial-to-trial variability.

The architecture of V1 exhibits an interesting combination of feedforward and recurrent connectivity: Neurons are distributed over several parallel two-dimensional (2D) sheets (Fig. 1A), commonly referred to in neuroscience as layers or laminae. The neurons are recurrently connected, but not randomly or in an all-to-all manner. Rather, synaptic connections exist primarily between nearby neurons, both within a layer and between layers. Connectivity between layers supports a strong feedforward stream of visual information from L4 to L2/3 to L5/6, which is complemented by a host of recurrent loops. The dominance of short connections makes it possible to combine in V1 extensive recurrent connectivity with a really small total wire length, which is essential for its physical realization.

The model of (*1*) also integrates, besides these anatomical details, a host of neurophysiological data about area V1. The point neuron version of this model that we are considering uses generalized leaky integrate-and-fire (LIF) neurons, more precisely GLIF_3_ neurons. These have, in addition to the membrane potential, two further hidden variables that model slower processes in biological neurons. The large diversity of neurons in the brain is reflected in the model of (*1*) through the use of 111 different types of GLIF_3_ neuron models that have each been fitted to experimental data in the Allen Brain Atlas (*2*).

The original model of (*1*) is not able to solve nontrivial computing tasks, because its synaptic weights were chosen on the basis of sparse experimental data about the mean and variance of synaptic weights. In contrast, synaptic weights in the living brain are individually tuned through a host of synaptic plasticity processes, and these processes induce higher-order correlations between weights that are crucial for computing capabilities of the network. At present, we do not have enough data about these plasticity processes to reproduce them in a model. However, we can address the question of what visual processing capabilities are supported by the model if synaptic weights are aligned for visual processing tasks through stochastic gradient descent. We applied this strategy to five different visual processing tasks that have commonly been considered in biological experiments (*3*–*8*). Afterward, our model achieved high accuracy simultaneously for all five tasks while working in a biologically realistic sparse firing regime close to criticality (*9*, *10*). Unexpectedly, its performance level remained in the same high-performance regime as the brain, even when we subjected the V1 model to noise in the images and in the network that it had not encountered during training. We demonstrate that this out-of-distribution (OOD) generalization capability of the V1 model with regard to new perturbations is far superior to that of convolutional neural networks (CNNs). We provide an explanation for that through an analysis of neural coding properties of these two types of models: Both use high-dimensional neural codes for images. However, the neural representation in the model of (*1*) is more robust because it uses, like the brain (*11*), a power law for the explained variance in higher principal components analysis (PCA) components that is close to a theoretically optimal compromise between the goal to be sensitive to details of visual inputs and the goal to be robust to noise from the visual input and within the network. In contrast, neural codes in CNNs were shown to have a different power law (*12*) that favors the first (coding precision) over the second goal (noise robustness). In addition, we demonstrate that the model of (*1*) preferentially uses those dimensions of population activity for coding that are orthogonal to the largest noise dimensions, like the brain does (*3*).

Together, our results show that the currently available anatomical and neurophysiological data, as compiled in (*1*), provide the basis for a new generation of neural network models for visual processing that can solve diverse visual processing capabilities in a highly robust manner. Furthermore, these neural network models provide new paradigms for neuromorphic computing because they combine versatility and robustness to noise with small total wire length and highly energy-efficient sparse activity.

## RESULTS

### Integration of anatomical and neurophysiological data, as well as data on noise in the brain, into a neural network model of area V1

Several decades of intense research efforts have accumulated a large body of knowledge about the anatomy and neurophysiology of the visual cortex, especially for primary visual cortex, i.e., for area V1. However, it has remained unknown to what extent this insight into the structure of V1 can be related to its function. We have examined this question for the case of the large-scale model for a patch of V1 in mouse from (*1*), which is arguably the most comprehensive integration of anatomical and neurophysiological data on area V1 that are currently available.

The network model is a spatially structured model for a patch of V1 that consists of 51,978 neurons from four main classes: one class of excitatory neurons and three classes of inhibitory neurons (Fig. 1B) that are distributed over five horizontal layers of neurons, labeled as L1, L2/3, L4, L5, and L6. Synaptic connections between these neurons are generated from data-based connection probabilities. These are defined in terms of base connection probabilities (Fig. 1C) that depend on the class and layer of the pre- and postsynaptic neuron. These base connection probabilities are scaled for each concrete pair of neurons by an exponentially decaying function of the lateral distance between their somata (Fig. 1D). This distance-dependent scaling entails that most synaptic connections are between nearby neurons and, hence, that the total wire length is small. However, it also affects the specific style of computational processing in the V1 model: Information is not continuously spread out all over the network as in randomly connected recurrent neural networks, which are frequently used as models for neural networks of the brain. To transform images and movies into input currents to neurons in this V1 model, we used the preprocessing module [lateral geniculate nucleus (LGN) model] of (*1*) (see Fig. 1A). It consists of 17,400 filters that model in a qualitative manner the responses of four classes of experimentally observed LGN neurons (sustained ON, sustained OFF, transient ON/OFF, and transient OFF/ON), which are further subdivided according to preferred temporal frequencies. We will refer in the following to this model of V1 in conjunction with the LGN model of (*1*) as the V1 model.

Individual neurons are modeled as point neurons. However, in contrast to the customary LIF neuron models, the V1 model uses 111 different variations of the LIF model, which are referred to as GLIF_3_ neuron models because they have, in addition to the membrane potential, two other internal variables that model after-spike currents in the neuron on slower time scales. These 111 different neuron types have been fitted to experimental data for 111 selected neurons from the neocortex according to the cell database of the Allen Brain Atlas (*2*).

The neurons in the model of (*1*) also received, besides inputs from the LGN model and inputs from other neurons, a small noise current. This noise was generated by a single Poisson source for all neurons. Hence, this noise is highly correlated, but its amplitude is so small that it has only little impact on neural firing (Fig. 1E). As such, we focused instead on a data-driven noise model. This noise model is based on experimental data from area V1 of the awake mouse (*11*). More precisely, we used the heavy-tailed distribution of noise amplitudes that arises from their experimental data (fig. S1). Furthermore, we superimposed two forms of noise: a quick form noise with scaling factor *q*, where a new value is drawn every millisecond from this distribution, mimicking, for example, noise that arises from stochastic synaptic release, and a slow form of noise with scaling factor *s*, where a new value is drawn from this heavy-tailed distribution once at the beginning of each trial. The latter mimics the well-known dependence of neural responses to the state of the network at the beginning of a trial [see, e.g., (*13*)]. We use the default values *s* = *q* = 2 for scaling these two forms of noise. The resulting noise model causes a qualitatively similar trial-to-trial variability of network responses in the V1 model as in the brain [compare fig. S2A with extended data figure 5 of (*11*)]. This trial-to-trial variability is shown for two sample neurons from the V1 model in Fig. 1F.

The resulting Fano factor of spike counts in 10-ms windows has then a value of 1.46 in the V1 model, which is close to the measured value of 1.39 in mouse V1 (*14*). To get a clearer picture of the noise robustness of the V1 model, we tested its computational performance also for substantially larger values of the scaling factors *q* and *s*.

### The V1 model can solve diverse computational tasks on visual input streams

**Fig. 2.**
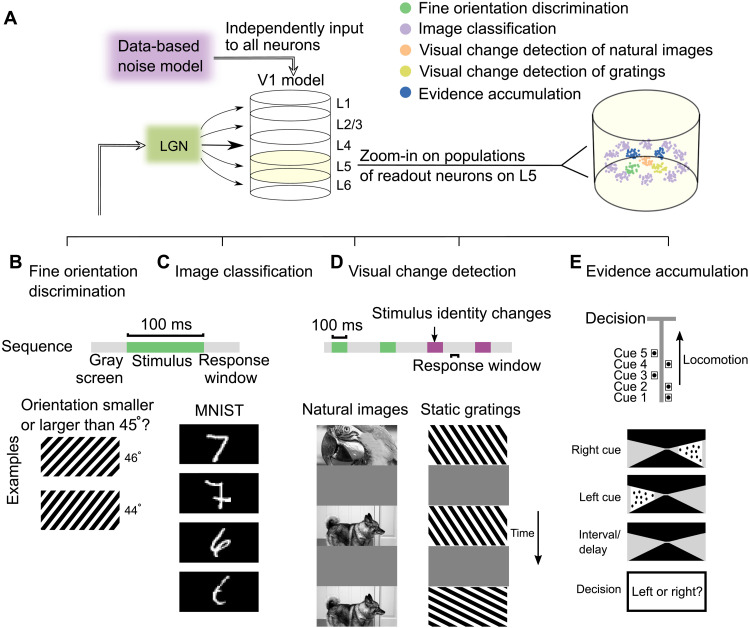
Illustration of the readout convention and the five visual processing tasks for which the V1 model was trained. (**A**) The visual stimuli for all five tasks were presented to the LGN model. Separate competing pools of pyramidal cells on L5 were chosen as readout neurons for each task. (**B** to **E**) Schematic diagrams and timings of five visual tasks (Materials and Methods). (B) In the fine orientation discrimination task, the network received a drifting grating with an orientation very close to 45°, and neurons in the corresponding readout pool had to fire if the orientation was larger than 45^∘^. (C) For the image classification task, the network received a handwritten sample of a digit from 0 to 9 from MNIST dataset, and the corresponding one of the 10 readout pools for this task had to fire stronger than the others (two samples for digits 7 and 6 are shown). (D) For the visual change detection task, a long sequence of images was presented, with gray screens in between. A corresponding readout pool had to become active during the response window if the most recent image differed from the preceding one. Both natural images and static gratings were used. (E) In the evidence accumulation task, seven cues were presented sequentially, and after a delay, a corresponding readout pool had to indicate through stronger firing whether most cues had been presented on the left or the right.

Classification of static images is a very popular test for neural networks. However, brains have visual processing capabilities that go far beyond that, because visual information arrives in natural environments, especially in the presence of active vision, in a piecemeal manner. Hence, brains need to be able to integrate temporally dispersed information, which can, in general, not be carried out by a feedforward neural network. Furthermore, brains can solve many different visual processing tasks with the same neural network without changing their synaptic weights. We wondered whether the V1 model also has this capability. Therefore, we tested it after training not only on a standard image classification task, classification of handwritten digits from the MNIST (modified national institute of standards and technology) database, but also on four tasks that require temporal integration of visual information (Fig. 2A). The latter ones have all been used in mouse experiments, and data on their behavioral performance are available. The five selected tasks are illustrated in Fig. 2: discrimination of subtle differences in the orientation of drifting gratings (Fig. 2B), as in the experiments of (*3*, *4*); a generic image classification task (Fig. 2C); visual change detection tasks for natural images and static gratings (Fig. 2D), as considered in (*5*, *6*); and accumulation of temporally dispersed cues on the left and right (Fig. 2E), as considered in (*7*, *8*), some of them with slightly longer delay periods.

To test the performance of the V1 model on these tasks, one has to specify a convention for extracting the network decision. One frequently used convention [see, e.g., (*15*)] is to let an external “readout neuron” that receives synaptic input from all neurons in the network produce the network decision (fig. S3A). Obviously, there are no such readout neurons in the brain that receive synaptic inputs from all neurons in a patch of the neocortex. Furthermore, this convention is not suitable for probing the computational capability of such a network model. Theoretical results (*16*, *17*) imply that if the network model is sufficiently large and has diverse units so that it approximately satisfies the point-wise separation property for relevant input streams, such global readout neurons become computationally quite powerful even if only the weights of these readouts are trained for a specific task. Hence, global linear readouts tend to mask the computational contribution of the neural network model itself. This theoretical prediction turns out to be valid also for the V1 model: If one takes the V1 model as defined in (*1*), without changing any of its synaptic weights or other parameters, and just trains linear readouts from all of its neurons for the five tasks, one already gets a very high average accuracy of all five tasks: 86.99%. Therefore, we used for the V1 model a more brain-like readout convention, where projection neurons within the network (*18*) extract and transmit computational results of the network. In particular, a large fraction of pyramidal cells on L5 projects computational results of the network to subcortical areas where behavioral responses are triggered. Consequently, we selected for each computational task and each possible outcome of a network decision a population of 30 excitatory neurons on L5 (Fig. 2A). If this population produced more spikes during the response window than competing populations that voted for other outcomes, then the outcome for which it “voted” was viewed as the network decision. The size of this readout population turned out to have no substantial impact (see Materials and Methods). It also caused no significant difference whether the neurons of each readout pool were colocated or are randomly distributed on L5 (fig. S3). Note that a colocated readout pool can possibly benefit from synergy, whereas a distributed pool can integrate information from more and more widely distributed presynaptic neurons. Hence, it is not a priori clear which works better. In our test, both versions perform about equally well. One important difference to the convention of using global readout neurons is that the set of neurons in the network that provides synaptic inputs to a neuron within the network is substantially smaller and spatially constrained.

With the values of synaptic weights provided by (*1*), the V1 model is incapable of performing any of the five tasks with this biologically more realistic readout convention: The accuracy is close to chance level (=42%). Even if one trains linear readouts from these small populations of neurons on L5 for the five tasks, one achieves in the case of the untrained V1 model an accuracy of just 53.74%. We therefore applied stochastic gradient descent, like in (*19*), to the synaptic weights of synapses within the V1 model of (*1*) and to connections from the LGN model to the V1 model. No synaptic connections were added or deleted during this process. We also made sure that the signs of synaptic weights could not change, i.e., we maintained the validity of Dale’s law. We used a loss function for gradient descent that penalized inaccurate decisions by the chosen populations of readout neurons (see Materials and Methods). The loss function also penalized biologically unrealistic high firing rates.

The principle of stochastic gradient descent is to let the network compute on a given input for one of the computational tasks, or in our case for a batch of such inputs for all five tasks. Then, the loss function is computed for these computations, and gradient descent is applied to the network computations to determine in which direction and by how much each synaptic weight should be changed to decrease the loss function. Then, the whole process is iterated. Stochasticity arises in this gradient descent from random selection of network inputs and is, in general, useful for avoiding getting stuck in local minima of the loss function. We applied stochastic gradient descent to the V1 model through a variation of backpropagation through time (BPTT), with the help of a suitable pseudo-derivative for handling the discontinuous dynamics of spiking neurons, as suggested by (*20*). To avoid artifacts arising from an application of gradient descent to the hard reset of GLIF_3_ neurons after a spike, we subtracted instead a fixed value from the membrane potential after each spike. Control experiments show that this modification of the neuron model causes no significant difference in the spike output of a neuron (fig. S4). The pseudo-derivative is just an approximation to ideal stochastic gradient descent, especially when gradients have to be propagated backward through several spikes. However, it appears to be the most powerful tool for training networks of spiking neurons that is currently available. It turns out to work especially well for the GLIF_3_ neuron models of (*1*), especially in a biologically realistic sparse firing regime, because gradients can be propagated with high precision through the slowly changing continuous variables of GLIF_3_ neuron models.

We found that the type of noise that is applied during training has little influence on the accuracy that is reached during testing: Virtually the same accuracy is achieved when during training the noise model from (*1*), our data-driven noise model, or no noise at all is applied while testing the data-driven noise model (see fig. S5). We think that this effect is due to the implicit noise that is introduced through the natural in-class variance of network inputs during training, because this variance is likely to dominate from the perspective of network function the impact of any milder form of noise within the network. We always report accuracy on test data for the case of data-driven noise within the network. Training was carried out for each network model over five different runs, two with the noise model of (*1*), two with the data-driven noise model (see Fig. 1F), and one run without any noise applied during training. Our motivation for also considering the noise model of (*1*) was to maintain comparability with their original model and for enabling an unbiased evaluation of the impact of various types of noise. After training, the V1 model achieved on all five tasks a performance that was in the same range as reported behavioral data (Table 1), with an average accuracy of 94.28%. Sample computations of the V1 model for each of the five tasks are shown in figs. S6 to S10. The performance after training did not depend on our particular choice of readout neurons in L5: Choosing randomly distributed instead of colocated pyramidal cells yielded an average accuracy of 94.67%. As expected, choosing instead global linear readout neurons for each task and training both the readout weights and the weights in the V1 model led to substantially higher accuracy of 98.13%. Hence, we find that the neural codes in the network could, in principle, support even better task performance, but biologically realistic readout from V1 reduces the amount of information that can be used by downstream networks for decision-making. This effect suggests an interesting new explanation for why behavioral performance of mice lags behind neural coding fidelity in area V1 (*4*) (note S1).

**Table 1.** The V1 model achieves high accuracy in all five tasks, consistent with the behavior performance of mice in similar tasks, after 16 training epochs. The chance level of performance in image classification task is 10%, and those in other tasks are 50%. Note that the behavioral experiments had longer time delays, which made the tasks somewhat more difficult.

	**Test accuracy**	**Behavior accuracy**	**Mean firing rate (Hz)**	**Spike raster**
Fine orientation discrimination	93.17%	∼83%^*^	3.97	Fig. S6
Image classification	92.11%	N/A	4.11	Fig. S7
Visual change detection of natural images	92.13%/92.14%^†^	∼73 % /77%^‡^	3.97	Fig. S8
Visual change detection of gratings	94.64%	∼60%^§^	3.90	Fig. S9
Evidence accumulation	99.32%	∼85%^ǁ^	3.96	Fig. S10

*Estimated from figure 4C of (*45*) when the orientation difference was 90°.

†In the visual change detection task for natural images, the two values refer to testing with familiar and novel images, respectively.

‡Estimated from figure 1I of (*5*) when familiar and novel images were presented to mice.

**Fig. 3.**
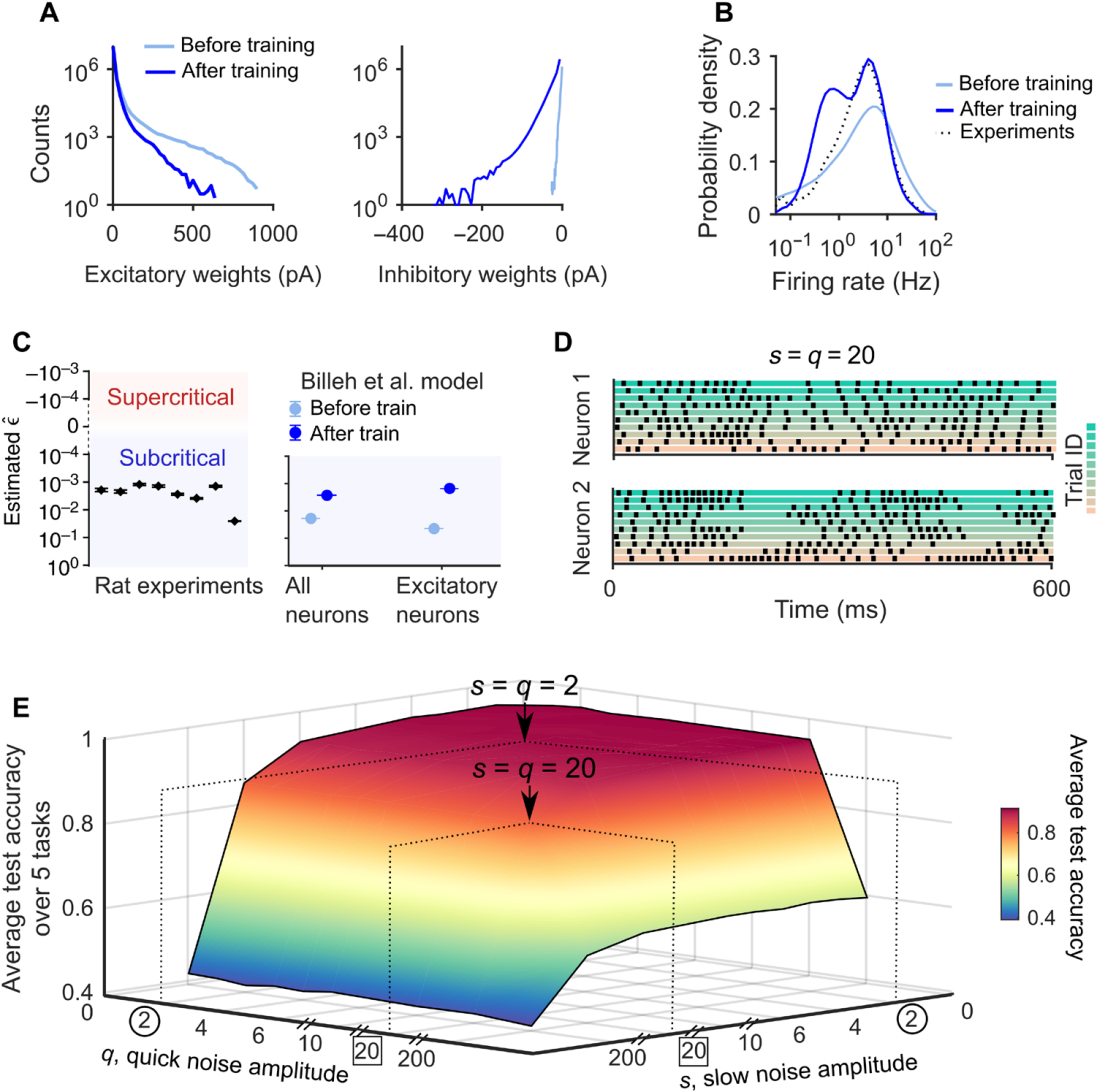
Analyses of the V1 model after training. (**A**) Distributions of excitatory weights (left) and inhibitory weights (right) before and after training. (**B**) Average distributions of firing rates for the five tasks before and after training. The distribution is moved through training closer to the one recorded in V1 (*6*). (**C**) Criticality of the V1 model is analyzed and compared with experimental data. The *y* axis shows estimates of $\hat{\epsilon }=1-\hat{m}$, where $\hat{m}$ is the estimated branching ratio. Estimates of this value in the rat brain from (*44*) are shown on the left. The V1 model produces almost the same branching ratio as the brain, especially after training. Error bars on the left represent 16 to 84% confidence intervals. Error bars on the right represent SEM over 10 trials. (**D**) The resulting trial-to-trial variability of neural firing is substantial when *s* = *q* = 20; see this panel for samples of spiking activity of the same two neurons as in Fig. 1 (E and F) for 10 trials with the same network input (image). (**E**) Average accuracy of the V1 model on test data for the five tasks, as a function of the amplitude *q* of quick noise and the amplitude *s* of slow noise. The model had been trained just with the default noise of (*1*), which has much less impact on neural activity according to Fig. 1 (E and F). The arrow in the back points to the accuracy for the default values *s* = *q* = 2 for the data-driven noise model. One sees that the average accuracy for the five tasks is also robust to much larger noise amplitudes, e.g., for *s* = *q* = 20; see arrow in front; it still has an average accuracy of 84.53%.

§Estimated from figure 3A of (*46*) when the orientation difference was 5°.

ǁEstimated from figure 1C of (*7*) when the number of cues was 6.

The median strength of inhibitory synapses increased from 0.03 to 4.80 pA during training, while the median weights of excitatory synapses decreased from 2.96 to 2.08 pA (see Fig. 3A and fig. S11 for more detailed analyses in terms of the neuron types involved). The distribution of neural firing activity was, after training, still close to the measured distribution in the brain (see Fig. 3B) with an average firing rate of 4 Hz. Furthermore, training preserves spatial clustering of orientation tuning in L2/3 (fig. S12), a neural coding feature that had been demonstrated experimentally for mouse V1 in (*21*).

Experimental data suggest that neural networks of the brain typically operate in a critical regime (*10*, *22*, *23*). We evaluated the criticality of the V1 model by measuring its branching ratio of neural activity, as suggested by (*10*). We found that both the untrained and the trained V1 models operate in a slightly subcritical regime. Training moved the model somewhat closer to the critical regime, reaching values of the branching ratio that almost perfectly matched recorded data from the brain (Fig. 3C). Hence, the V1 model operated in a dynamic regime that closely matches experimental data.

We tested the robustness of the resulting versatile visual processing capability of the V1 model after training by exposing its neurons to substantially larger amplitudes *q* and *s* of the data-driven noise model (see Fig. 3D as an example), although it had never been exposed to such noise during training [where we only applied the really small noise considered in (*1*)]. Unexpectedly, it is almost impossible to destroy its versatile visual processing capability (Fig. 3E): It remained stable even when the amplitudes *q* and *s* of quick and slow noise were increased by several orders of amplitude.

### Impact of anatomical and neurophysiological details of the V1 model on learning speed and resulting computational performance

**Fig. 4.**
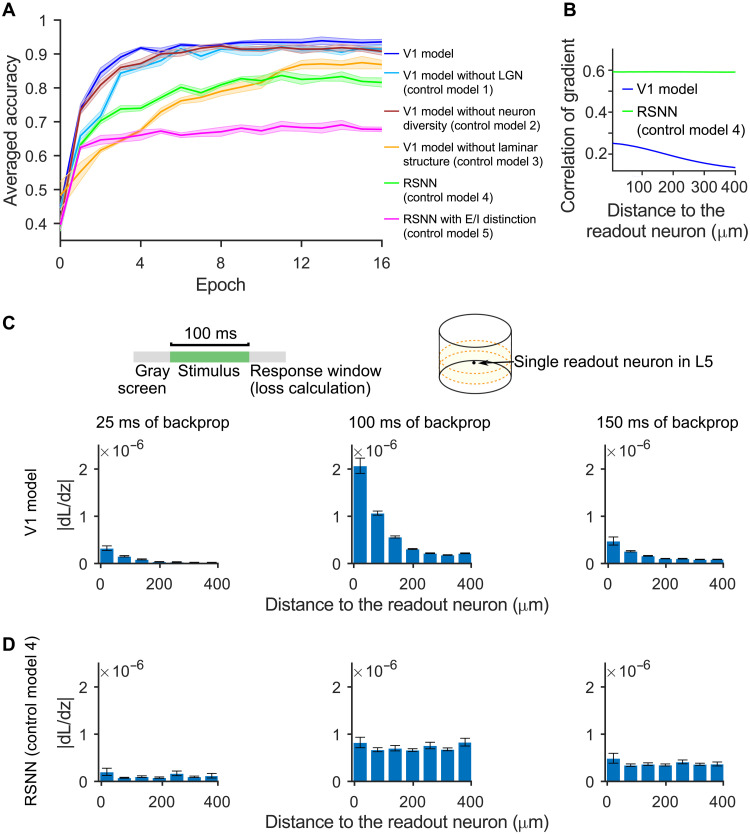
Impact of details of the V1 model on learning speed and resulting computational performance. (**A**) Accuracy on test examples of the V1 model and of five control models after varying numbers of training epochs. Whereas neuron diversity and the inclusion of the LGN model have only moderate impact on the learning speed and resulting performance, deletion of the laminar structure drastically reduced learning speed and notably reduces the performance level that can be reached. RSNN control models have similar deficiencies, with further reduction in achievable performance. Shaded areas represent the SEM over five runs. (**B**) Resulting correlation of total gradient information between neuron pairs in the V1 model and a randomly connected control model 4 (RSNN), collected during a full backpropagation pass (600 ms) of the loss function used in the visual change detection task of natural images. Nearby neurons in the V1 model receive correlated gradient information, which supports coherent learning in local network modules. (**C** and **D**) Spread of gradient information from a single readout neuron in L5 to other neurons depends on their distance, shown after 25, 100, and 150 ms of backpropagation from the readout neuron. Whereas these learning signals primarily reach nearby neurons in the V1 model, they are uniformly spread out all over the network in the RSNN. The loss function is the one used in the image classification task, but applied here only to a single readout neuron in the center of L5.

We show in Fig. 4A the performance of the V1 model and of five control models on test data, in dependence of the training duration. In control model 1, we removed the LGN model of (*1*) and injected pixel values directly into the V1 model, targeting the same neurons as the LGN model. This turns out to have just a minor effect on the properties that we have analyzed. In control model 2, we removed the diversity of the 111 data-based neuron types in the V1 model, replacing them with one generic model for excitatory neurons and one for inhibitory neurons. In control model 3, we removed instead the laminar spatial structure with distance-dependent connection probabilities of the V1 model, replacing them with an equal number of randomly chosen connections (without dependence on spatial proximity). Control model 4 is a randomly connected recurrent network of standard LIF neurons [randomly connected networks of spiking neurons (RSNN)] with the same number of neurons and connections. This is arguably the most commonly considered type of spiking neural network model. Control model 5 is a variation of it, where the neurons are split like in the V1 model into excitatory and inhibitory neurons, and Dale’s law is preserved during training. This type of model is sometimes seen as an intermediate step from generic RSNNs toward more biologically oriented network models. Two remarkable phenomena are demonstrated in Fig. 4A:

#### (i) The V1 model learns much faster than control models, reaching close to optimal performance after just four training epochs

A closer look at training progress for individual tasks (fig. S13) shows that the V1 model makes progress on each task in a continuous and smooth manner, whereas control models tend to reach early plateaus from which they can only escape after very long further training.

The laminar structure with primarily local synaptic connections of the V1 model also affects the dynamics of stochastic gradient descent: Gradients that result from errors at a readout neuron are not immediately spread out all over the network, which happens in generic RSNNs, but are rather propagated in a wave-like manner over larger and larger parts of the network (Fig. 4, C and D). Consequently, correlations between learning gradients have, for two neurons in the V1model, a more pronounced dependence on their distance than in a randomly connected control model (Fig. 4B). Hence, the V1 connectivity structure better supports localized computation and learning within a large network.

With regard to the weight initialization before training, we show in fig. S14 that if one replaces the weight values of the V1 model from (*1*), which were used as weight initialization in all of the previously described experiments, with a random initialization that is commonly used in neural network studies, this drastically reduces performance after the first five training epochs but later provides the same accuracy.

#### (ii) The V1 model reaches higher accuracy than the control models, even when each control model is trained much longer

Removal of the laminar structure (control model 3) reduces the accuracy more than the removal of neuron diversity (control model 2). However, both of these control models still reach higher accuracy than the standard RSNN (control model 4). Last, partitioning the neurons of the standard RSNN into excitatory and inhibitory neurons (control model 5), which could be seen as an interesting interpolation between the most abstract RSNN model and the V1 model, reduces performance in comparison to the generic RSNN.

### The power spectrum of neural codes provides an explanation for the astounding robustness of visual processing by the V1 model

**Fig. 5.**
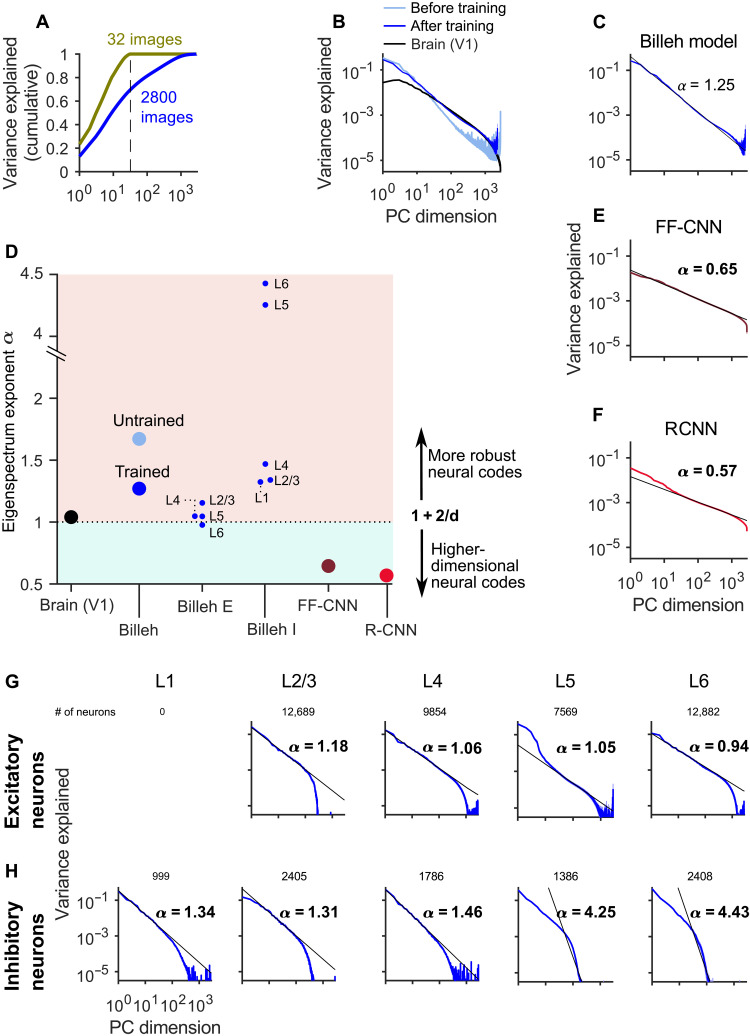
Comparing the eigenspectrum of neural codes in the V1 model of (*1*) with data from the brain and with CNNs. (**A**) As in the brain, the cumulative fraction of explained variance saturates only at the dimension of the input ensemble, here shown for *d* = 32 and 2800 natural images. (**B**) Eigenspectra of the untrained/trained V1 model of (*1*) with an ensemble of 2800 randomly chosen natural images, and for mouse V1 (*11*). (**C**) Exponents of the power law for the V1 model of (*1*), all for the same ensemble of 2800 randomly drawn natural images. (**D**) Summary result for exponents of the power law for the V1 model of (*1*) and for CNNs. Billeh E and Billeh I mark results where the eigenspectrum is computed for specific populations of excitatory and inhibitory neurons of the model of (*1*). (**E** and **F**) Same as in (C) but for feedforward CNNs (FF-CNNs) and recurrent CNNs (RCNNs), respectively. (**G**) Eigenspectra of excitatory neurons on different layers of the V1 model (*1*) exhibit values close to the measured value from a large sample of neurons in V1. (**H**) Eigenspectra of inhibitory neurons on different layers.

An explanation for the robustness of visual processing in area V1 has been provided by (*11*). They verified through large-scale recordings from V1 in mouse a theoretically predicted link between noise robustness of visual processing and neural codes for images. They found that V1 uses high-dimensional neural codes for images, but the power of higher PCA components decays sufficiently fast so that its neural codes remain noise robust. More precisely, they introduced a cross-validated PCA that provides unbiased estimates of the stimulus-related variance. They found that the amount of explained variance continues to increase as further PCA dimensions were included without saturating below the dimensionality (= size) *d* of the image ensemble. We applied exactly the same analysis to the trained model of (*1*) and found that the model exhibited the same coding property (Fig. 5A). It was also shown in (*11*) that the explained variance of the *n*th principal component of network representations of images follows a power-law *n*^−α^. The exponent α characterizes how fast the variance that is explained by higher PCA dimensions decays. Their theoretical analysis predicts that α = 1 + 2/*d* is the optimal value, because this value provides a theoretically optimal compromise between encoding too many details (leading to smaller values of α) and keeping the neural code robust to perturbations (leading to larger values of α). Other theoretical work (*24*) also predicts that a value of α close to 1 enhances under mild conditions downstream generalization performance. In vivo recordings of (*11*) found that the value of α for primary visual cortex of mouse is close to this optimal value α = 1 + 2/*d*. This neural coding property of area V1 in the brain has gained additional interest through the contrasting result of (*12*). They found that feedforward CNNs (FF-CNNs), which are viewed to be substantially less noise robust than the brain, have in fact a smaller α, as predicted by the theory. Hence, we wondered whether our more brain-like neural network model for visual processing would exhibit a value that is closer to the theoretical optimum. We applied for that purpose the same measurement procedure as (*11*) to the V1 model, for a set of 2800 randomly drawn natural images. Figure 5B shows the eigenspectrum of PCA component for the model of (*1*) before and after training, and also the measured eigenspectrum of V1 responses from (*11*). One sees that the eigenspectrum of the model is already, before training, quite close to that of the brain and is moved by training even closer. The resulting exponent α of the power law (Fig. 5C) is for the model somewhat higher than in the brain. Figure 5 (D, G, and H) suggests that this is largely due to the contributions of inhibitory neurons. They are generally found to have less precise neural codes for sensory stimuli, and consistent with that, their eigenspectra decayed substantially faster in the V1 model.

We have also reproduced the result of (*12*) that FF-CNN has a substantially smaller value of α (Fig. 5E) and found that the recurrent CNN (RCNN) model of (*25*), which also achieves very high accuracy for image classification, has an even smaller value of α (Fig. 5F). Together, our results imply that the V1 model has, unlike CNNs, similar neural coding properties as area V1 in the brain. Furthermore, these can be linked, according to the theory of (*11*), to its remarkable noise robustness.

### Comparing noise robustness and OOD generalization of the V1 model and CNNs

**Fig. 6.**
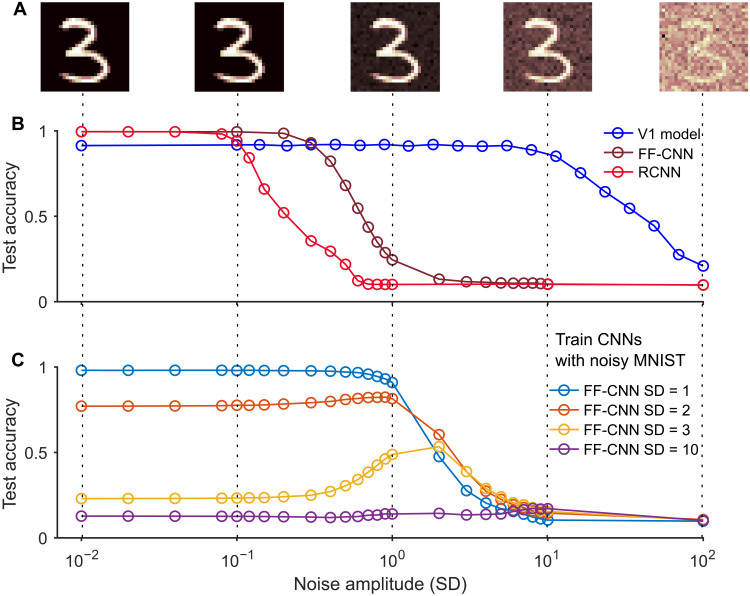
Robustness of the V1 model and of CNNs to noise in images. (**A**) Samples of handwritten MNIST digits with Gaussian noise drawn independently from 𝒩(0, SD) for each pixel, for different values of the SD. (**B**) While the V1 model never quite reaches the peak performance of the CNNs, it tolerates noise with fairly high SD, whereas the performance of FF-CNNs and RCNNs is substantially degraded even by noise with small SD. (**C**) Even when CNNs are trained on images with particular noise statistic (SD), they do not generalize well to test images with a different value of SD. Furthermore, they do not achieve for SD between 1 and 10 the same noise robustness as the V1 model even when they were trained on images with that type of noise.

Our preceding analyses of neural coding in the V1 model of (*1*) and CNNs suggest that the former is more noise robust. Because it is hard to compare their robustness to noise within the networks with that of CNNs, and because their computational units are so different, we compared instead their robustness to noise in the visual input, concretely to Gaussian pixel noise that was added to handwritten digits from the MNIST dataset. We used Gaussian noise with mean 0 and different SD. We first trained each type of neural network on the original dataset without noise and then tested their classification performance on images with noise (see Fig. 6A for samples). Figure 6B shows that the classification performance of the V1 model is substantially more robust to the added noise during testing, as predicted by the preceding analysis of the different neural coding strategies of the V1 model and CNNs. To check whether CNNs become more noise robust when they are, like the V1 model, subjected to internal noise during training, we applied dropout (*26*) to them: We randomly replaced tensor outputs of ReLu layers with probability *p* with 0. It turns out that the robustness of CNNs is not improved substantially by that (see the results for various values of *p* in fig. S15).

Because neither of these networks had been trained with the noisy images for which they were tested, we analyzed here a particular OOD generalization capability of the V1 model and of CNNs. Figure 6C demonstrates that even if CNNs are trained with a particular noise statistics, they do not perform well if they are tested on images with a different SD of Gaussian noise. In contrast, the V1 model exhibited perfect OOD generalization in this respect. In addition, the remarkable robustness of the V1 model to noise within the network (Fig. 3E), even if no noise had not been present during training (fig. S5B), can be viewed as an OOD generalization capability.

### Neural coding dimensions for visual inputs are in the trained V1 model largely orthogonal to noise, like in the brain

**Fig. 7.**
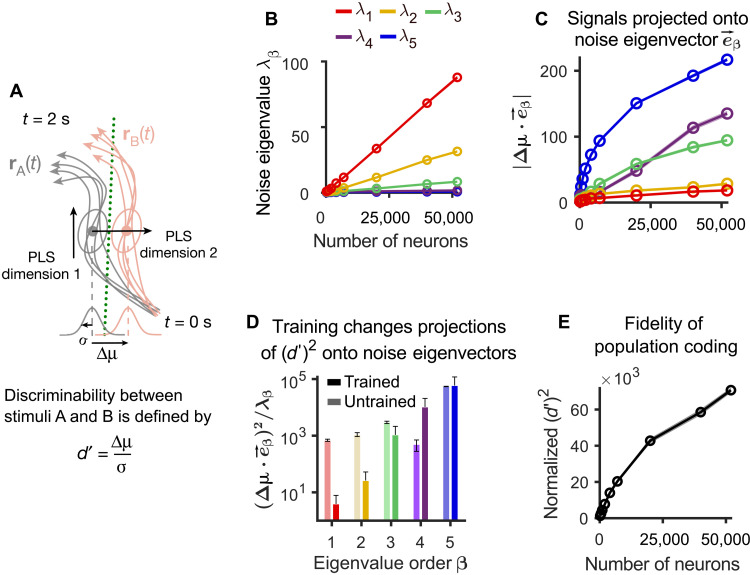
Analyses of relations between signaling and noise dimensions in the V1 model and of the impact of noise correlations when one considers larger numbers of neurons. (**A**) Schematic of the calculation of the discriminability index *d*′ according to (*3*). (**B**) The five largest eigenvalues λ_β_ (β = 1,2, ⋯,5) of the noise covariance matrix in the trained V1 model increase linearly with the number of sampled neurons. (**C**) Neural coding dimensions (Δμ) in the trained V1 model were nearly orthogonal to the dominant eigenvectors of the noise covariance matrix, ${\vec{e}}_{\alpha }$, invariant to neuron numbers. (**D**) Training moved the neural coding dimensions so that they became more orthogonal to the dominant noise dimensions. (**E**) The squared discriminability index (*d*′)^2^ kept increasing when applied to more neurons of the V1 model. *d*′ values were normalized by those obtained for trial-shuffled data (averaged across 1 s). Shaded areas in (B), (D), and (E) and error bars in (C) represent the SEM over 100 trials.

A further explanation for robust coding capabilities of the brain was provided by experimental data of (*3*). They reported that dimensions in which differences between visual stimuli were encoded in area V1 of mouse, i.e., neural coding dimensions, were nearly orthogonal to the largest noise modes. This result provided evidence that neural coding in V1 was even robust to noise. In other words, cortical design principles appear to enhance coding robustness by restricting most noise fluctuations in area V1 to dimensions of the population activity that do not impede neural coding of visual inputs. We wondered whether the V1 model would inherit this important property. Therefore, we carried out the analysis of (*3*) also for the V1 model, using the same visual stimuli (moving gratings with orientation differences that were close to the perception threshold). The discriminability index *d*′ from (*3*) is a measure that they proposed as a measure for the fidelity of neural population coding (see the illustration in Fig. 7A). Neural population responses **r**_A_(*t*) and **r**_B_(*t*) to two stimuli A and B form two distributions (ellipses). Partial least square (PLS) analysis projects them onto a subspace where they become most distinct. The discriminability *d*′ is defined as the separation, Δμ, of the two distributions along the dimension orthogonal to the optimal boundary (green line) for classifying stimuli in this subspace, divided by the SD of each distribution along this dimension.

The eigenvalues of the noise covariance matrix are plotted in Fig. 7B as a function of the number of neurons that are sampled in the V1 model. We found that, also in the V1 model, the projection of the signal difference Δμ onto the eigenvectors for the largest noise eigenvalues is relatively small (Fig. 7C). Furthermore, compared with the untrained V1 model, training of this model moved the signaling dimensions to become more orthogonal to the largest noise dimension (see Fig. 7D). The projection of (*d*′)^2^ on an eigenvector can be interpreted as the signal-to-noise ratio because it is the ratio of the projected signal difference and noise eigenvalue (Materials and Methods).

It had been argued in (*3*) that the amount of visual information that is encoded in area V1 reaches a ceiling due to noise correlations. This result appeared to contradict the results of (*11*). A possible explanation of this discrepancy was offered in (*4*): They conjectured that the seemingly limited coding capability of V1 could be explained by the relatively small number of up to 1300 neurons from which simultaneous recordings had been carried out in (*3*). They suggested that the apparent “ceiling” would rise if one records from more neurons. We tested this hypothesis in the V1 model of (*1*) and found that the corresponding measurement *d*′ for the total amount of encoded information keeps increasing, although at a somewhat slower rate, when the number of neurons from which one records in the model rises from 1300 to 51,978 (see Fig. 7E).

### Concrete anatomical and neurophysiological features of the V1 model that are responsible for its superior noise robustness

**Fig. 8.**
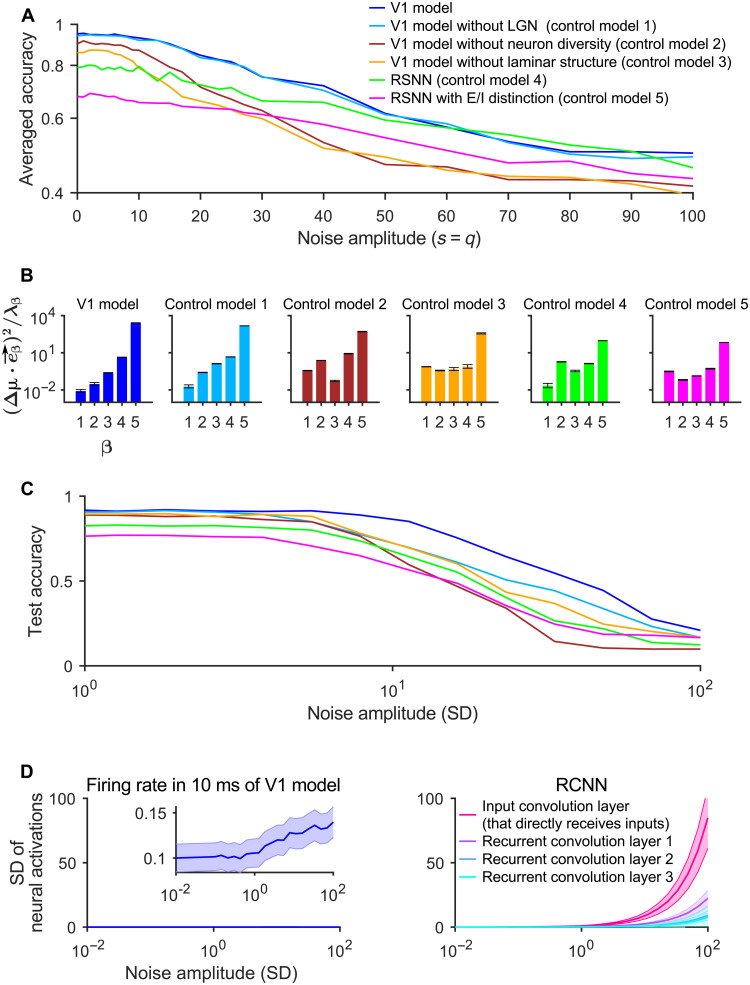
Concrete factors of the V1 model that are responsible for its superior noise robustness. (**A**) Decay of accuracy of the V1 model and five control models for increasing amplitudes of internal noise (see Fig. 3E for a preceding analysis of this for the V1 model). One sees that both neuron diversity and laminar structure are important factors for the robustness of the V1 model to internal noise. (**B**) Orthogonality of neural coding and noise dimensions in the V1 model (see Fig. 7D) and five control models after training. Only control model 1 (V1 model without LGN) has similarly small projections onto the noise eigenvectors with the largest eigenvalues, i.e., for small values of β. Neuron diversity and laminar structure turn out to be important factors for that. (**C**) Decay of accuracy of the V1 model and five control models for image classification (MNIST task) with increasing amount of noise in the visual input. Neuron diversity and laminar structure of the V1 model turn out to be also essential for the superior robustness of the V1 model to noise in the input. Color scheme is the same as in (A). (**D**) Analysis of the impact of the same noise as in (C) on neurons in the V1 model and in the RCNN of (*25*). When the SD of the pixel noise becomes larger than 1, which is the range where the performance of the RCNN becomes substantially worse than that of the V1 model according to Fig. 6B, the output of RCNN units becomes substantially stronger affected by this input noise than the firing activity of neurons in the V1 model. Shaded areas in left and right represent 0.1-fold SD and SD across neurons, respectively. Inset of left panel shows the same data but for a different order of magnitude.

We had shown in Fig. 3E that the V1 model is robust to rather high levels of internal noise. Figure 8A demonstrates that the laminar spatial organization of the V1 model and its diversity of neuron types are both important factors for that. This is corroborated by the analysis of the discriminability index *d′* for control models, where also the projection of neural codes onto the noise dimensions with the largest eigenvectors assumes substantially larger values for the corresponding control models 2 and 3 than for the V1 model (Fig. 8B). Figure 8C shows that the laminar spatial organization and diversity of neuron types of the V1 model are also both major factors for the robustness of the V1 model to external noise in visual inputs (compare with Fig. 6). Furthermore, in comparison with CNNs, neurons in the V1 model are affected much less by this external noise (see Fig. 8D). We hypothesize that a major factor for that is the substantially larger in-degree of neurons in the connectivity graph of the V1 model (on average, 278) compared with a CNN (*9*), which enables them to integrate over a much larger number of image pixels, thereby making their output less dependent on noise in individual pixels. This is insofar interesting, because the small indegree of stereotypical CNN units is a cornerstone of the CNN architecture. The control models also exhibited a power spectrum of neural codes, like the V1 model (Fig. 5). The exponent α of the power spectrum was further away from the ideal value 1 + 2/*d* than that of the V1 model (fig. S16), suggesting that these control models are operating in a less desirable regime. However, their α values were larger than that of the V1 model. This would predict better noise robustness according to the underlying theory, but this theoretical prediction is inconsistent with our previously discussed empirical analysis.

The LGN model also contributes to the robustness of internal noise. As shown in Fig. 8A, control model 1 (where the LGN model is removed) is less robust to external noise than the V1 model with the LGN. Consistent with that, adding this LGN model (without further training of its parameters) as preprocessor to the RCNN markedly improves its noise robustness (fig. S17). Hence, the spatiotemporal filters of this LGN model can filter out some of the noise, which is consistent with the preceding results of (*27*).

## DISCUSSION

We have demonstrated that the V1 model of (*1*), which arguably provides the largest currently available accumulation of anatomical and neurophysiological data on area V1 in the mouse brain, provides a window not only into brain dynamics but also into visual processing capabilities and neural coding properties that are entailed by these data. In particular, we found that this V1 model exhibits interesting advantages with regard to learning speed and visual processing performance over models that are closer to common artificial neural network models, such as RSNNs and CNNs. Furthermore, we have isolated concrete anatomical and neurophysiological features of the V1 that are responsible for that.

CNNs are currently most commonly used for visual processing in artificial intelligence (AI). They were also inspired by some aspects of visual processing in the brain, especially the existence of simple and complex cells in area V1. However, a closer look shows that they differ from V1 in the brain in almost all other respects: with regard to their computational units (artificial neurons in CNNs versus spiking neurons in the brain), the diversity of their units (very few simple units versus a large diversity of neurons with different temporal dynamics), their large-scale architecture (usually feedforward, versus recurrent with laminar structure), their small-scale architecture (very simple network motifs versus a complex combination of feedforward and recurrent processing in cortical microcircuits), and total wire length (almost quadratic versus just linear growth with the number of processing neurons). V1 in the brain, however, also differs from CNNs with regard to two important visual processing capabilities: The brain is more versatile because it can solve a number of diverse visual tasks with the same synaptic weights, in particular also tasks that require integration of sequentially arriving visual information. In addition, visual processing in the brain is very noise robust, also to new types of noise (OOD generalization). We have shown here that the previously listed fundamental differences between the structure of V1 in the brain and CNNs are causally related to these two superior visual processing capabilities of the brain: The V1 model of (*1*), which integrates a large body of experimental data on area V1 in the brain, is able to perform similarly versatile and robust visual processing. Furthermore, we could identify concrete anatomical and neurophysiological features of the V1 model that are responsible for this.

Because we can now reproduce these two important functional capabilities of area V1 in a model, we have a new research platform at our disposal for studying how neural coding properties of the brain emerge from its anatomical and neurophysiological features and how they are related to its visual processing capabilities. We have demonstrated here the feasibility of this new research strategy by applying it to the V1 model, an analysis of its neural coding that had already been used successfully for elucidating neural codes for images in area V1 of the brain: We analyzed the eigenspectrum of the explained variance of principal components of its neural codes for images. We found that the listed structural features of V1 in the brain do in fact induce a salient feature of its neural coding strategy: The V1 model exhibits a similar power law for neural codes of images as the brain. In contrast, applying the same training process to CNNs, CNNs exhibit a power law with a substantially slower decay of the eigenspectrum. According to the theoretical analysis of (*11*), this implies that neural codes in CNNs are less noise robust. By regularizing CNNs (*28*) or training them with different methods (*24*), one can move the exponent of their eigenspectra closer to 1 + 2/*d*, which can improve the robustness of CNNs. We have also shown that the anatomical and neurophysiological data on V1 that have been integrated into the V1 model suffice to reproduce a further salient aspect of neural coding in area V1 of the brain, where, according to (*3*), about 90% of the noise fluctuations in area V1 are constrained to dimensions of the population activity that are orthogonal to noise dimensions. We found that this is an emergent property of the V1 model of (*1*).

On a more general level, we have shown that the V1 model of (*1*) can be seen as the first prototype of a new generation of neural network models for visual processing that capture substantially more features of brain processing than CNNs. Further work is needed to tease apart the functional implications of each of its structural features and to port similar advanced brain-like visual processing capabilities into simpler neural network models. Often one uses, instead of data-based models for neural networks of the brain, randomly connected recurrent networks of strongly simplified neuron models. In our experience, neural coding and computational properties of recurrent neural networks vary substantially in dependence on their connectivity structure and neuron models. This highlights the need to test brain-like features not only in abstract models but also in neural network models that integrate our available knowledge about the actual structure of these neural networks in the brain.

Our method can also be applied to investigate more detailed models of V1, e.g., models that include data on the lattice of microcolumns of neurons in L5 (*29*) and on short-term plasticity (*30*) and functionally salient aspects of dendritic spikes (*31*). It also provides a paradigm for elucidating how anatomical and neurophysiological details of interconnected higher and lower brain areas carry out distributed computations, in particular how interaction of neurons in superficial layers of V1 with higher cortical areas enhances visual processing capabilities of V1. Although we have focused here on readout neurons in L5, the sparsely firing pyramidal cells in L2/3 of the trained V1 model contain already most of the information that is needed to solve the computational tasks of the network, and hence, they could potentially transmit this to higher areas: Readouts from these neurons (which might be seen as proxies for neurons in higher cortical areas that receive input from V1) can be trained to solve all five tasks with high accuracy (fig. S18). A substantial body of anatomical and neurophysiological data on higher cortical areas and their connectivity to V1 is currently available for that [see, e.g., (*6*, *32*, *33*)]. We expect that deficits in visual processing capabilities of the V1 model, such as limited spatial integration of image features and a relatively short working memory time span, will disappear when the V1 model is combined with models of higher brain areas. In addition, neurons on L2/3 of the V1 model will then be placed into a biologically more realistic context, where they send computational results to higher areas and receive inputs from them.

The analysis of neural coding in the V1 model has produced a number of predictions for future biological experiments. In particular, we have shown in Fig. 7E that correlated noise reduces the coding fidelity of the network but does not produce an a priori bound for its sensory discrimination capability [this had already been hypothesized by (*4*)]. A further prediction of our model is that the PCA eigenspectrum of neural codes for inhibitory neurons does not obey a power law for higher dimensions (see Fig. 5H). Last, the values of excitatory synaptic weights in V1 are predicted to generally shrink through training (Fig. 3A, left), while inhibitory weights are predicted to become stronger (Fig. 3A, right).

Visual processing in the brain exhibits also with regard to two aspects of physical implementation two attractive features: Most synaptic connections in V1 are between nearby units, which is essential for an efficient physical realization of synaptic connections in neuromorphic hardware. This architectural feature is also likely to support faster learning (Fig. 4A). In addition, computations are carried out in V1 through event-based processing with very sparse firing activity. This computing regime not only is very energy efficient but also supports computations on tasks where temporal aspects play an important role, because it allows to let time represent itself in network computations. Since we have shown that both of these features can be reproduced in a corresponding neural network model for visual processing, this V1 model suggests that it will also be possible to recruit them for the design of substantially more energy-efficient neuromorphic implementations of visual processing (*34*).

## MATERIALS AND METHODS

### Neuron models

We based our study on the “core” part of the point-neuron version of the realistic V1 model introduced by (*1*). To make it gradient friendly, we replaced the hard reset of membrane potential after a spike emerges with the reduction of membrane potential *z_j_*(*t*)(*v_th_* − *E_L_*), where *z_j_*(*t*) = 1 when neuron *j* fires at time *t* and *z_j_*(*t*) = 0 otherwise. *v_th_* is the firing threshold of membrane potential. *E_L_* is the resting membrane potential. This causes no substantial change in the neural response (fig. S4). We simulated each trial for 600 ms. The dynamics of the modified GLIF_3_ model was defined as

$$\begin{array}{c} v_{j}(t+\delta t)=\\ \alpha v_{j}(t)+\frac{1-\alpha \tau }{C}(I_{j}^{e}(t+1)+\sum_{m}I_{j}^{m}(t+1)+{\mathrm{gE}}_{L}+I_{j}^{\text{syn}}(t))-z_{j}(t)(v_{\text{th}}-E_{L})\\ z_{j}(t)=H(v_{j}(t)-v_{\text{th}})\\ I_{j}^{e}(t)=\sum_{i}W_{\mathrm{ji}}^{\text{in}}x_{i}(t)+{\mathrm{qK}}_{j}^{\text{quick}}(t)+{\mathrm{sK}}_{j}^{\text{slow}} \end{array}$$ (1)

where *C* represents the neuron capacitance, *I*^e^ is the external current, *I*^syn^ is the synaptic current, *g* is the membrane conductance, and *v*_th_ is the spiking threshold. $W_{\mathrm{ji}}^{\text{in}}$ is the synaptic weight from LGN neuron *i* to V1 neuron *j*. The scales of the quick noise $K_{j}^{\text{quick}}(t)$ and the slow noise $K_{j}^{\text{slow}}$ to neuron *j* are *q* = 2 and *s* = 2, respectively, unless otherwise stated. *K_j_* was randomly drawn from the empirical noise distribution, which will be elaborated on later. The decay factor α is given by *e*^−δ*t*/τ^, where τ is the membrane time constant. δ*t* denotes the discrete-time step size, which is set to 1 ms in our simulations. *H* denotes the Heaviside step function. To introduce a simple model of neuronal refractoriness, we further assumed that *z_j_*(*t*) is fixed to 0 after each spike of neuron *j* for a short refractory period depending on the neuron type. The after-spike current *I^m^*(*t*) was modeled as

$$I^{m}(t+\delta t)=f^{m}I^{m}(t)+z(t)\delta I^{m};m=1,\ldots ,N_{\text{asc}}$$ (2)

where the multiplicative constant *f^m^* = exp (−*k^m^*δ*t*) and an additive constant, δ*I^m^*. In our study, *m* = 1 or 2. Neuron parameters have been fitted to experimental data from 111 selected neurons according to the cell database of the Allen Brain Atlas (*2*) [see (*1*, *35*)], including neuron capacity *C*, conductance *g*, resting potential *E*_L_, the length of the refractory period, as well as amplitudes δ*I^m^* and decay time constants *k^m^* of two types of after-spike currents, *m* = 1,2.

### Synaptic inputs

The V1 model specifies the connection probability between neurons based on experimental data. The base connection probability for any pair of neurons from the 17 cell classes is provided in (*1*) by a table (shown in Fig. 1C); white grid cells denote unknown values. The entries in this table are based on measured frequencies of synaptic connections for neurons at maximal 75-μm horizontal intersomatic distance. This base connection probability was scaled by an exponentially decaying factor in terms of the horizontal distance of the somata of the two neurons (Fig. 1D). This distance-dependent scaling is also based on statistical data from experiments (leaving aside finer details of connection probabilities). The synaptic delay was spread in [1,4] ms, which was extracted from figure 4E of (*1*) and converted to integers as the integration step is 1 ms.

The postsynaptic current of neuron *j* was defined by the following dynamics (*1*)

$$I_{j}^{\text{syn}}(t+\delta t)=e^{-\frac{\delta t}{\tau_{\text{syn}}}}I_{j}^{\text{syn}}(t)+\delta te^{-\frac{\delta t}{\tau_{\text{syn}}}}C_{j}^{\text{rise}}(t)$$ (3)

$$C_{j}^{\text{rise}}(t+\delta t)=e^{-\frac{\delta t}{\tau_{\mathrm{syn}}}}C_{j}^{\text{rise}}(t)+\sum_{i}W_{\mathrm{ji}}^{\text{rec}}z_{i}(t)\frac{e}{\tau_{\mathrm{syn}}}$$ (4)

where τ_syn_ is the synaptic time constant, $W_{\mathrm{ji}}^{\text{rec}}$ is the recurrent input connection weight from neuron *i* to *j*, and *z_i_* is the spike of presynaptic neuron *i*. The τ_syn_ constants depend on neuron types of pre- and postsynaptic neurons (*1*).

### Initial conditions

The initial conditions of spikes and membrane potentials were zero unless stated otherwise. The initial conditions of **W**^in^ and **W**^rec^ were given by the values in (*1*) unless stated otherwise.

### Data-driven noise model

The noise currents $K_{j}^{\text{quick}}(t)$ and $K_{j}^{\text{slow}}$ in Eq. 1 were randomly drawn from an empirical noise distribution. The quick noise $K_{j}^{\text{quick}}(t)$ was drawn independently for all neurons in every 1 ms; the slow noise $K_{j}^{\text{slow}}$ was drawn independently for all neurons once 600 ms. The empirical noise distribution (fig. S1) was from the additive noise decoded from experimental data of mice response to 2800 nature images (*11*). The decoding method was cross-validation PCA (cvPCA) (*11*), which will be elaborated later. It measures the reliable variance of stimulus-related dimensions, excluding trial-to-trial variability from unrelated cognitive and/or behavioral variables or noise. We collected the variability (additive noise) to form the empirical noise distribution. We refer to the methods and supplementary materials of (*11*) for a detailed mathematical analysis of this method.

### Readout populations

By default, we used 15 readout populations in the V1 model, whose firing activity during the response window encoded the network decisions for the five visual processing tasks. Each population consisted of 30 randomly selected excitatory neurons in L5, located within a sphere of a radius of 55 μm, with some distance between these spheres for different readout populations (fig. S3B). The results were not sensitive to the number of neurons in each population. We tested populations consisting of 15 and 60 readout neurons and found that the performance difference is less than 0.2%. We had also shown in (*19*) that picking just two neurons from the trained readout pool to produce network outputs provided almost the same accuracy for the visual change detection task.

We also considered the case where the neurons in these readout populations were randomly distributed in L5 (fig. S3C), and the case where each population was replaced by global linear readout neurons, which received synaptic inputs from all neurons with activity (**Z**) in the V1 model, i.e., **Y**_global_ = **W**_readout_**Z** + **B**, **B** is the bias (fig. S3A).

### Visual processing tasks

We designed details of these five tasks to be as close as possible to corresponding biological experiments while keeping them as simple as possible. Only for the image classification task (MNIST), no corresponding mouse experiments exist.

#### LGN model

The visual stimuli were preprocessed by the LGN model (Fig. 2A) according to (*1*) (it is actually meant to model preprocessing by the retina and LGN in a qualitative manner). This LGN model consists of 17,400 spatiotemporal filters that model responses of LGN neurons in mouse to visual stimuli (*36*). Each filter produces a positive output that is interpreted as firing rates of a corresponding LGN neurons.

According to the requirements of this LGN model, each visual input pixel was first converted to gray scale and scaled into an interval [−*Int*, *Int*], *Int* > 0. The output of the LGN model was injected into the V1 model as external currents, i.e.,

$$I_{\text{sti}}=W^{\text{in}}\cdot \text{LGN}(G_{\mathrm{Int}})$$ (5)

where *G_Int_* represents images scaled into [−*Int*, *Int*] for *Int* = 2.

#### Fine orientation discrimination task

In mouse experiments, mice were trained to distinguish orientation of drifting grating stimuli (*3*, *4*). The stimuli were presented for 750 ms or longer. To reproduce this task under the limitations of graphics processing unit GPU memory, we input drifting grating to the V1 model through the LGN model for 100 ms (Fig. 2B). As in (*4*), stimuli were sinusoidal drifting gratings (spatial frequency, 0.05 cycles per degree; drifting rate, 2 Hz). In both the training and testing processes, the orientation was uniformly drawn from [43,47]^∘^ (i.e., 45 ± 2) with the precision of 0. 1^∘^. The orientation difference was the same as in (*4*). The initial phase was randomly sampled. The simulation sequence included 50-ms delay, 100-ms drifting gratings, and 50-ms response window in order.

In the response window, we defined the mean firing rate of readout population as

$$r_{\text{readout}}=\frac{1}{T_{\text{resp}}\cdot N_{\text{readout}}}\sum_{t=1}^{T_{\text{resp}}}\sum_{j=1}^{N_{\text{readout}}}z_{j}(t)$$ (6)

where the sum over *j* is over the *N*_readout_ = 30 readout neurons and the sum over *t* is over the time length of response window *T*_resp_ = 50 ms. If *r* > *r*_0_ = 0.01, then this reported a network decision that the orientation was larger than 45^∘^. Otherwise, it reported that the orientation was smaller than 45^∘^.

#### Image classification task

To demonstrate that the V1 model is also able to classify images, we included the task to classify handwritten digits from 0 to 9 from the MNIST dataset (Fig. 2C). The timing of input images and response windows was the same as in the preceding task. The task was to decide which digit was denoted by the handwritten image (two samples for 7 and 6 are shown in Fig. 2C). Each of the 10 readout populations for this task was assigned to one of the 10 digits. The network decision was taken to be that digit for which the readout population fired most strongly during the response window.

#### Visual change detection task with natural images

In mouse experiments (*5*, *6*), mice were trained to perform the visual change detection task with natural images. A sequence of static natural images (250 ms), interleaved by short phases (500 ms) of gray screens, was presented as visual input; mice had to report whether the most recently presented image was the same as the previously presented one. To reproduce this task under the limitations of GPU memory, we presented natural images for 100 ms each, with the gray delays between them lasting for 200 ms (Fig. 2D). Note that the first image was presented after 50 ms. All images were selected from a set of 40 randomly chosen images from the ImageNet dataset (*37*). The probability that the next image differed from the preceding one was set to 50%. In case of a changed image identity, the model had to report within a time window of 50 ms length that started 150 ms after image onset (response window). If the mean firing rate of the readout population in the response window *r*_readout_ > *r*_0_, it reported a network decision that the image had changed. The computation of the V1 model on this task has been further analyzed in (*19*).

#### Visual change detection task with drifting gratings

We also replaced the natural images above with static gratings that have different orientations and kept the input sequence the same (Fig. 2D). The setting of the static grating is the same as in the fine orientation discrimination task, except that it is static. The changing probability of orientation is 50%; the orientation of static gratings was uniformly drawn in [120,150] (i.e., 135 ± 15) with the precision of 0. 1^∘^.

#### Evidence accumulation task

A hallmark of cognitive computations in the brain is the capability to go beyond a purely reactive mode: to integrate diverse sensory cues over time and to wait until the right moment arrives for an action. A large number of experiments in neuroscience analyze neural coding after learning such tasks [see, e.g., (*7*, *8*)]. We considered the same task that was studied in the experiments of (*7*, *8*). There, a rodent moved along a linear track in a virtual environment, where it encountered several visual cues on the left and right (Fig. 2E). Later, when it arrived at a T-junction, it had to decide whether to turn left or right. The network should report the direction from which it had previously received most visual cues. To reproduce this task under the limitations of a GPU implementation, we used a shorter duration of 600 ms for each trial. The right (left) cue was represented by 50 ms of cue image in which the black dots on the right (left) side of the maze. Visual cues were separated by 10-ms delay, and cues were represented by the gray wall of the maze. After a delay of 250 ms, the network had to decide whether more cues had been presented on the left or right, using two readout populations for left and right. The decision was indicated by the more vigorously firing readout pool (left or right) within the response window of 50 ms.

### Loss function

The loss function was defined as

$$L=L_{\text{cross}-\text{entropy}}+\lambda_{f}L_{\text{rate}\;\text{reg}.}+\lambda_{v}L_{v\;\text{reg}.}$$ (7)

where *L*_cross − entropy_ represents the cross-entropy loss and λ*_f_* and λ*_v_* represent the weights of firing rate regularization *L*_rate reg._ and voltage regularization *L*_v reg._, respectively. As an example, the cross-entropy loss of visual change detection tasks was given by

$$\begin{array}{c} L_{\text{cross}-\text{entropy}}=-\sum_{m}[T^{\left(m\right)}\text{log}\;\sigma (\theta (r_{\text{readout}}^{\left(m\right)}-r_{0}))+\\ \left(1-T^{\left(m\right)})\text{log}\;\sigma (\theta (r_{0}-r_{\text{readout}}^{\left(m\right)}))\right] \end{array}$$ (8)

where the sum over *m* is organized into chunks of 50 ms and $r_{\text{readout}}^{\left(m\right)}$ denotes the mean readout population firing rate defined in Eq. 6. Similarly, *T*^(*m*)^ denotes the target output intime window *m*, being 1 if a change in image identity should be reported and otherwise 0. The baseline firing rate *r*_0_ was 0.01. σ represents the sigmoid function. θ is a trainable scale (θ > 0) of firing rate.

We also used regularization terms to penalize unrealistic firing rates and unrealistic membrane voltages. Their weights, λ_f_ = 0.1 and λ_v_ = 10^−5^. The rate regularization is given by the Huber loss (*38*) between the target firing rates, *y*, calculated from the model in (*1*), and the firing rates, *r*, sampled the same number of neurons from the network model

$$\begin{array}{c} L_{\text{rate}\;\text{reg}.}=\sum_{j}^{N}|\tau_{j}-I\{\delta_{j}<0\}|\frac{L_{\kappa }(\delta_{j})}{\kappa },\;\text{with}\\ L_{\kappa }(\delta_{j})=\{\begin{array}{cc} \frac{1}{2}\delta_{j}^{2}, & \text{if}\;|\delta_{j}|\leq \kappa \\ \kappa (|\delta_{j}|-\frac{1}{2}\kappa ), & \text{otherwise} \end{array} \end{array}$$ (9)

where *j* represents neuron *j*, *N* represents the number of neurons, τ*_j_* = *j*/*N*, δ = 0.002, and $\delta_{j}=r_{j}-r_{j}^{\text{target}}$. I(x)=1 when *x* is true; I(x)=0 when *x* is false.

The voltage regularization was given by

$$L_{v\;\text{reg}.}=\frac{1}{N}\sum_{j=0}^{j=N}{\left({\left[\frac{v_{j}-E_{L}}{E_{L}}-1\right]}^{+}\right)}^{2}+{\left({\left[-\frac{v_{j}-E_{L}}{E_{L}}+1\right]}^{+}\right)}^{2}$$ (10)

where *N* represents the total number of neurons, *v_j_* represents the membrane potential of neuron *j*, *E*_L_ represents the resting membrane potential, and [⋯]^+^ represents rectifier function.

### Training and testing

We trained the model for all five tasks together. Pairs of visual inputs and target outputs were collected in separate 64 batches for each task, and these batches were interlaced during training. Apart from the change detection tasks, the spikes and membrane potentials were reset to 0 after each trial that consisted of 600 ms.

We applied BPTT (*19*) to minimize the loss function. The nonexisting derivative $\frac{\partial z_{j}}{\partial v_{j}}$ was replaced in simulations by a simple nonlinear function of the membrane potential that is called the pseudo-derivative. Outside of the refractory period, we chose a pseudo-derivative of the form

$$\begin{array}{c} \psi^{t}=\frac{\gamma_{\mathrm{pd}}}{v_{\mathrm{th}}-E_{L}}\text{exp}(\frac{-{\left(v_{\mathrm{sc}}^{t}\right)}^{2}}{\sigma_{p}^{2}}),\\ v_{\mathrm{sc}}^{t}=\frac{v^{t}-v_{\mathrm{th}}}{v_{\mathrm{th}}-E_{L}} \end{array}$$ (11)

where the dampening factor γ_pd_ = 0.5 and the Gaussian kernel width σ_p_ = 0.28. During the refractory period, the pseudo-derivative was set to 0.

We drew a batch of visual stimuli (in our case, batch size is 320, consisting of 64 network inputs for each of the five tasks) and calculated the gradient after every trial for each synaptic weight whether an increase or decrease of it (but without changing its sign) would reduce the loss function. Weights were then updated by the average gradient across the batch. This method had originally only been applied to neuron networks with differentiable neuron models and was normally referred to as stochastic gradient descent.

During the training, we added the sign constraint on the weights of the neural network to keep Dale’s law. Specifically, if an excitatory weight was updated to a negative value, it would be set to 0; vice versa.

In every training run, we used a different random seed to draw fresh noise samples from the empirical distribution and for randomly generating/selecting training samples. We would like to emphasize that we tested the trained model—whenever possible—for new visual stimuli that had not been shown during training (this was not possible for the gratings because there were not sufficiently many different visual stimuli for them). In that sense, we evaluated the generalization capability of the trained V1 model, rather than its capability to handle a fixed set of stimuli correctly (which often suffices to solve behavioral tasks in experiments). The model achieved on all five tasks a performance that is in the same range as reported behavioral data from corresponding mouse experiments (Table 1).

### Other simulation details

The BPTT training algorithm was coded, as the simulation of the model, in TensorFlow, which runs very efficiently on GPUs and also on multiple GPUs for training in parallel. We used independent simulations in parallel by distributing trials for all five tasks over batches. Every batch consisted of 320 trials, 64 for each of the five tasks. In every trial, the model of (*1*) was simulated for 600 ms of biological time, which took, together with the calculation of gradients, around 5 s on an NVIDIA A100 GPU. Once all batches had finished (one step), gradients were calculated and averaged to update the weights by BPTT. We define an epoch as 781 iterations/steps because this represents one cycle through the full training dataset of MNIST. This computation had to be iterated for 16 epochs to make sure the average performance on the five tasks was saturated. This took 60 hours of wall clock time on 160 GPUs.

### Control models

We used five control models in Fig. 4. In control model 1, we removed the LGN model and directly injected pixel values of the image into the V1 model. The image was resized to a number of pixels that roughly matched the number of LGN channels (2544). The pixel values were scaled by a factor 0.04 to bring the resulting firing activity into a reasonable regime. In control model 2, we removed the diversity of the 111 data-based neuron types in the V1 model, replacing them with one generic model for excitatory neurons (the excitatory neuron on L2/3, node type ID in Allen Brain Atlas: 487661754) and one for inhibitory neurons (PV neuron on L2/3, node type ID: 484635029). In control model 3, we removed instead the laminar spatial structure with distance-dependent connection probabilities of the V1 model, replacing them with an equal number of randomly chosen connections. Control model 4 is a randomly connected recurrent network of standard LIF neurons (RSNN) with the same number of neurons and connections as the V1 model. The membrane constant of these LIF neurons is 10 ms, and their refractory period is 5 ms long. These are common values for spiking neural network models. Control model 5 is a variation of this RSNN, where excitatory and inhibitory neurons are distinguished like in the V1 model, and Dale’s law is observed during training. This type of model is sometimes seen as an intermediate step from generic RSNNs toward more biologically oriented network models. The detailed settings are shown in Table 2. All control models were trained in the same way as the V1 model, i.e., for the same tasks and loss function, including the same sparsity regularization term and using the same hyperparameters for training.

**Table 2.** The control model settings.

**Model name**	**Neuron model**	Connectivity^*^	**Neuron type^†^**	**Weight sign constraint**	**Input via LGN model**
V1 model	GLIF_3_	V1	V1	Yes	Yes
V1 model without LGN (control model 1)	GLIF_3_	V1	V1	Yes	No
V1 model without neuron diversity (control model 2)	GLIF_3_	V1	1E 1I	Yes	Yes
V1 model without laminar structure (control model 3)	GLIF_3_	Random	V1	Yes	Yes
RSNN (control model 4)	LIF^‡^	Random	Single	No	Yes
RSNN with E/I distinction (control model 5)	LIF	Random	Single	Yes	Yes

*V1: the connectivity in the V1 model; random: randomly selected connectivity but the number of connectivity is fixed as in the V1 model.

**Table:** 

†V1: The diverse neuron types as in the V1 model (in total 111 types); 1E 1I: all excitatory neurons are the same (the excitatory neuron on L2/3, node type ID in Allen Brain Atlas: 487661754), and all inhibitory neurons are the same (PV neuron on L2/3, node type ID: 484635029); single: all neurons are the same.

‡LIF represents standard LIF neuron model.

### Branching ratio as a measure for criticality

On the basis of the work of (*39*), where the branching ratio was recommended as a rather reliable measure for criticality of a network, we examined this branching ratio for the V1 model. In particular, this measure was shown there to be more robust to subsampling. The branching ratio is defined as the ratio of the number of neurons spiking at time *t* + 1 to the number of spiking neurons at time *t*. Critical regimes, by their nature, are balanced and avoid runaway gain (positive or negative) and have a branching ratio of 1.0. We stimulated the V1 model as in the visual change detection task of nature images for 15 s.

In a network with *A* active neurons at time *t*, if the branching ratio has a fixed value *m*, then ⟨*A*_*t*+1_ ∣ *A_t_*⟩ = *mA_t_* + *h*, where < ∣ > denotes the conditional expectation, *m* is the branching ratio, and *h* is a mean rate of an external drive/stimulus. Considering subsampling, *a_t_* is proportional to *A_t_* on average < *a_t_*∣*A_t_*⟩ = η*A_t_* + ξ, where η and ξ are constants. This subsampling leads to a bias: *m*(η^2^Var [*At*]/Var [*at*] − 1). Instead of using time *t* and *t* + 1, this method focuses on times *t* and *t* + *k* with different time lags *k* = 1, …, *k*_maximum_. With this, the branching ratio *m_k_* is <*a*_*t*+*k*_ ∣ *a_t_* > = *m_k_* = η^2^Var [*A_t_*]/Var [*a_t_*]*m^k^* = *bm^k^*, where *b* is a constant. To compute *m_k_* with different *k*, we obtained an exponential curve and extracted *m* from this curve. *m* < 1 indicates a subcritical regime; *m* > 1 indicates a supercritical regime; *m* = 1 indicates a critical regime.

### Convolutional neural networks

#### Feedforward CNN

We used ResNet-18 (*40*) as FF-CNN. To calculate its eigenspectra, we used the pretrained version on ImageNet provided by PyTorch. To evaluate its robustness to pixel noise, we trained ResNet-18 on MNIST with the Adadelta optimizer. The batch size was 64; learning rate was 1; weight decay was 0.0001; the coefficient used for computing a running average of squared gradients was 0.9; the term added to the denominator to improve numerical stability was 1 × 10^−6^; and the number of training epochs was 10.

#### Recurrent CNN

We used the gated RCNN (*25*), inspired by abundant recurrent connections in the visual systems of animals. The gates control the amount of context information inputted to the neurons. We used the code GRCNN-55 (weight sharing). To calculate its eigenspectra, we used the pretrained version on ImageNet provided by (*25*). To evaluate its robustness to pixel noise, we trained RCNN on MNIST with the stochastic gradient descent optimizer. The batch size was 64; the learning rate was 0.1; the momentum was 0.9; the weight decay was 0.0001; and the number of training epochs was 10.

#### Adding dropout to CNN training

To introduce internal noise to the FF-CNN and the RCNN during training, we independently set outputs of ReLu units with probability *p* to 0 using samples from a Bernoulli distribution. This procedure is known as dropout training (*26*).

### Eigenspectrum analysis

#### Cross-validation PCA

Eigenspectra of V1 model were estimated by the explained variance of the neural response along with the *n*th principal component (computed from the first presentation). It is achieved by cvPCA that computes the covariance of the projections of neural responses for the two repeats onto this component. cvPCA measures the reliable variance of stimulus-related dimensions, excluding trial-to-trial variability from unrelated cognitive and/or behavioral variables or noise. It accomplishes this by computing the covariance of responses between two presentations of an identical stimulus ensemble (fig. S2B). Because only stimulus-related activity will be correlated across presentations, cvPCA provides an unbiased estimate of the stimulus-related variance. Briefly, the algorithm operates as follows

$$\begin{array}{cc} X^{\left(1\right)}= & {\mathrm{USV}}^{⊤}\;(\text{singular value decomposition})\\ {\tilde{X}}^{\left(1\right)}= & X^{\left(1\right)}V\;(\text{project data onto eigenvectors})\\ {\tilde{X}}^{\left(2\right)}= & X^{\left(2\right)}V\\ \lambda_{j}= & \sum_{i=1}^{s}{\tilde{X}}_{\mathrm{ij}}^{\left(1\right)}{\tilde{X}}_{\mathrm{ij}}^{\left(2\right)},\;\text{for}\;j\in \{1,\ldots ,C\}\;(\text{compute eigenvalue}) \end{array}$$

where ***X***^(1)^, ***X***^(2)^ ∈ ℝ^*S* × *N*^ (*S* is the number of stimuli, and *N* is the number of neurons) are the neural responses for the first and second half of the trials (and averaged across trials), ***V*** ∈ ℝ^*N* × *C*^ are the *C* eigenvectors of the covariance matrix of ***X***^(1)^, and λ ∈ *ℝ^C^* are the cross-validated eigenvalues associated with each of the eigenvectors (λ_***j***_ is the ***j***th eigenvalue).

The first step of the cvPCA algorithm computes the eigenvectors of the neural response covariance from one set of the trials. The second and third steps project the neural responses from each half of the trials onto each eigenvector. The final step computes the (scaled) variance of the neural responses when projected onto an eigenvector (that was computed using one-half of the trials). Thus, each cross-validated eigenvalue is related to the amount of stimulus-related variance of the neural responses along the eigenvalue’s corresponding eigenvector.

To be consistent with (*11*), we summed up spikes over 500 ms in response to visual stimuli. We ran cvPCA 10 times on the response of the neural network fed with the same images that are used in (*11*). On each iteration, we randomly sampled the population responses of each stimulus from the two repeats without replacement. We ran 10 different runs and found that they were very similar to each other, i.e., the SD was close to 0. For the trained V1 model, we calculated the eigenspectra in three models trained with different noise and randomly generated data, and found that the SD is 5.95 × 10^−5^. The displayed eigenspectra of the trained V1 model were averaged over these three models. The code is publicly available (*11*).

#### V1 model

We analyzed the neural representation in the trained V1 model in the same way as responses of V1 neurons were analyzed in (*11*): Without loss of generality, we used 2800 generic images that were randomly drawn from ImageNet validation dataset in all panels of Fig. 5. We also tried the 2800 nature images used in (*11*) and found that they gave rise to a slower decaying speed of eigenspectrum (1.15); they were only used in fig. S2A to compare with the noise level in mouse V1 experiment. We also used a smaller set of 32 images, repeated 90 times. All stimuli were input 50 ms after the simulation onset and sustained for 500 ms in each trial to be the same as experimental procedures. They were presented twice to allow cross-validated analysis. The initial condition of membrane potentials and spikes was set to zeros, unless otherwise stated. We input the 2800–nature image stimuli five times with different random seeds that were used to draw the noise and initial conditions of membrane potential and after-spike current from uniform distributions. We found that the results were not sensitive to the initial condition and noise.

#### Convolutional neural networks

Generic images were resized so that their shorter dimension was 256 pixels and then center-cropped to 224 × 224 pixels. Padding the image and resizing it to 224 × 224 pixels achieved similar results. Images were additionally preprocessed by normalizing each image channel (Red Green Blue channels) using the mean and SDs that were used during model training (mean, 0.485, 0.456, and 0.406; SD, 0.229, 0.224, and 0.225). For gray images, we repeated it to three channels. Using these preprocessed images, we extracted activation from every layer of each CNN and computed their eigenspectra using PCA because artificial neural responses are deterministic.

#### Power-law fitting of eigenspectra

Using the least-squares method, we fit power laws to the eigenspectra, *f*(*n*), against PC dimension, *n*. The fitting function is *f*(*n*) = *n*^−α^(*n* ∈ [*n*_min_, *n*_max_]), where *n*_min_ and *n*_max_ are lower and higher bounds, respectively. For most cases, we chose *n*_min_ ∈ [1,20] and *n*_max_ ∈ [301,2800]. For the 32-grating recordings, owing to noise and the length ofthe spectrum, we chose *n*_min_ ∈ [1,10] and *n*_max_ ∈ [14,35]. For each possible pair of *n*_min_ and *n*_max_, we estimated the exponent α and its goodness-of-fit by the coefficient of determination (*R*^2^). We then selected the estimate of *n*_min_, *n*_max_, and α that gave the maximum *R*^2^ (>0.99) over all possibilities.

### Discriminability index *d*′ for neural responses to visual stimuli

To estimate how much information the neural activity conveyed about the stimulus identity, following (*3*), we used the metric *d*′, which characterizes how readily the distributions of the neural responses to the two different sensory stimuli can be distinguished (*41*). The quantity (*d*′)^2^ is the discrete analog of Fisher information (*42*).

In Fig. 7 (A and E), to be consistent with the experimental study (*3*), we calculated the neural response as the spike counts in each bin of 200 ms and evaluated two different approaches to compute *d*′ values for the discrimination of the two different visual stimuli (gratings in the fine orientation discrimination task); the difference between two gratings is 2°; each stimulus was presented in 500 trials. We analyzed the neural responses in a specific time bin relative to the onset of visual stimulation, which was called as instantaneous decoding approach used in (*3*). The alternative way, cumulative decoding, i.e., analyzing neural responses that were concatenated over time from the start of the trial up to a chosen time, demonstrated similar results. To determine *d*′ accurately despite having about fewer trials than neuron number in the V1 model, we reduced dimension by using PLS analysis (*43*) to identify and retain only five population vector dimensions in which the stimuli were highly distinguishable as in (*3*). In this 5D representation, the neural dynamics evoked by the two stimuli become distinguishable over the first 200 ms of stimulus presentation. In the reduced space, we calculated the (*d*′)^2^ value of the optimal linear discrimination strategy as

$${\left(d\prime \right)}^{2}=\Delta \mu^{T}\Sigma^{-1}\Delta \mu =\Delta \mu^{T}w_{\text{opt}}$$ (12)

where $\Sigma =\frac{1}{2}(\Sigma_{A}+\Sigma_{B})$ is the noise covariance matrix averaged across two stimulation conditions, Δμ = μ_A_ − μ_B_ is the vector difference between the mean ensemble neural responses to the two stimuli, and ***w***_opt_ = Σ^−1^Δμ, which is normal to the optimal linear discrimination hyperplane in the response space (*42*). Each entry of a covariance matrix is the covariance of spike counts of two neurons *a_i_* and *b_i_* (*i* ∈ {1,2, ⋯, *N*}, *N* is the number of trials): $\frac{1}{N-1}\sum_{i=1}^{N}(a_{i}-\bar{a})(b_{i}-\bar{b})$, where $\bar{x}$ is the mean of {*x_i_*}.

We also calculated ${\left(d_{\text{shuffled}}^{\prime }\right)}^{2}$, the optimal linear discrimination performance using trial-shuffled datasets, which we created by shuffling the responses of each cell across stimulation trials of the same stimulus. Owing to this shuffling procedure, the off-diagonal elements of Σ_A_ and Σ_B_ became near zero. ${\left(d_{\text{shuffled}}^{\prime }\right)}^{2}$ increased much faster with the increase of sampled neurons than did (*d*′)^2^.

In Fig. 7B, noise eigenvalue λ_β_ and its eigenvector ${\vec{e}}_{\beta }$ were calculated by eigendecomposition of noise covariance matrix Σ. In Fig. 7C, to quantify the signals projected onto the eigenvector ${\vec{e}}_{\beta }$, we projected Δμ onto ${\vec{e}}_{\beta }$ and calculated its norm $|\Delta \cdot \mu {\vec{e}}_{\beta }|.$ In Fig. 7D, to demonstrate that training makes signaling dimensions more orthogonal to the largest noise dimension, we decompose (*d*′)^2^ into a sum of projections

$${\left(d\prime \right)}^{2}=\Delta \mu^{T}\Sigma^{-1}\Delta \mu =\sum_{\beta }(\frac{{|\Delta \mu \cdot {\vec{e}}_{\beta }|}^{2}}{\lambda_{\beta }})$$ (13)

Because $\Delta \mu \cdot {\vec{e}}_{\beta }$ corresponds to the signal projected on noise eigenvector ${\vec{e}}_{\beta }$ and noise eigenvalue λ_β_ corresponds to the noise scale on ${\vec{e}}_{\beta }$, $\frac{{|\Delta \mu \cdot {\vec{e}}_{\beta }|}^{2}}{\lambda_{\beta }}$ can be interpreted as singal-to-noise ratio on eigenvector ${\vec{e}}_{\beta }$. The eigenvectors well aligned with Δμ are the most important for discriminating between the two distributions of neural responses.

## Supplementary Materials

### This PDF file includes:

Table S1 Figs. S1 to S18 Supplementary Note 1

## Notes

View/request a protocol for this paper from *Bio-protocol*.
