# Supplementary material for: A data-based large-scale model for primary visual cortex enables brain-like robust and versatile visual processing
Source: Sci Adv. 2022 Nov 2;8(44):eabq7592. doi: 10.1126/sciadv.abq7592 (PMC9629744; doi:10.1126/sciadv.abq7592)
Supplement: Supplementary file 1 — Table S1 Figs. S1 to S18 Supplementary Note 1 [file sciadv.abq7592_sm.pdf]

Science Advances
AAAS

Supplementary Materials for

A data-based large-scale model for primary visual cortex enables brain-like robust and versatile visual processing

Guozhang Chen *et al.*

Corresponding author: Wolfgang Maass, maass@igi.tugraz.at

Sci. Adv. **8**, eabq7592 (2022)
DOI: 10.1126/sciadv.abq7592

This PDF file includes:

Table S1
Figs. S1 to S18
Supplementary Note 1

Table S1: Performances on the 5 tasks are not very sensitive to the amplitude of internal noise in the network. The noise level used in this study is $q = 2$, $s = 2$. All tasks were tested on testing dataset.

	Fine orientation discrimination	Image classification	Visual change detection of nature images	Visual change detection of gratings	Evidence accumulation
$q = 1$, $s = 0$	94.66%	91.92%	93.30%	94.44%	99.20%
$q = 1$, $s = 2$	94.47%	92.11%	92.83%	94.64%	99.56%
$q = 1$, $s = 3$	94.18%	91.96%	92.48%	93.96%	99.48%
$q = 1$, $s = 4$	94.01%	91.77%	92.99%	94.68%	99.56%
$q = 2$, $s = 2$	93.17%	92.11%	92.13%	94.64%	99.32%
$q = 3$, $s = 3$	94.46%	91.66%	92.99%	94.56%	99.28%
$q = 3$, $s = 4$	94.61%	91.84%	92.32%	94.60%	99.28%
$q = 4$, $s = 3$	94.69%	91.50%	92.85%	93.40%	99.24%
$q = 4$, $s = 4$	94.39%	91.94%	92.43%	94.24%	99.20%
$q = 5$, $s = 4$	94.63%	92.08%	92.31%	95.12%	99.52%
$q = 10$, $s = 10$	92.27%	90.34%	88.29%	92.64%	97.20%
$q = 20$, $s = 20$	83.57%	84.39%	75.30%	90.96%	88.96%
$q = 200$, $s = 200$	45.79%	10.55%	50.52%	48.24%	49.08%

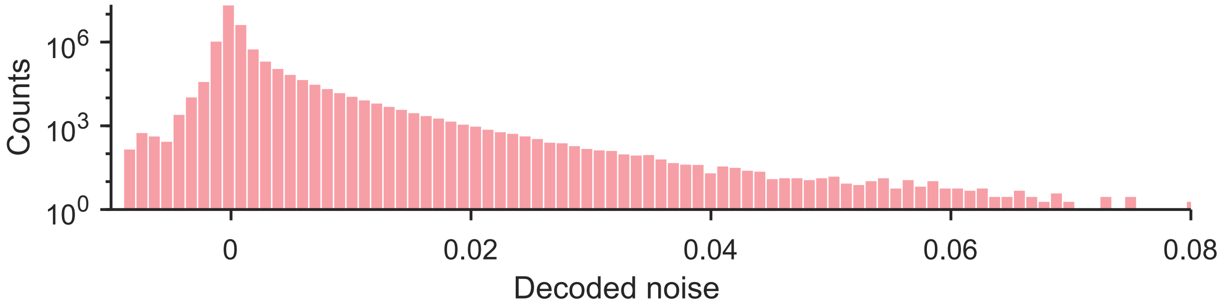

Figure S1: Empirical noise distribution from which noise values for our data-driven noise model are drawn. They are based on experimental data from (*11*) (see Data-driven noise model in Materials and Methods).

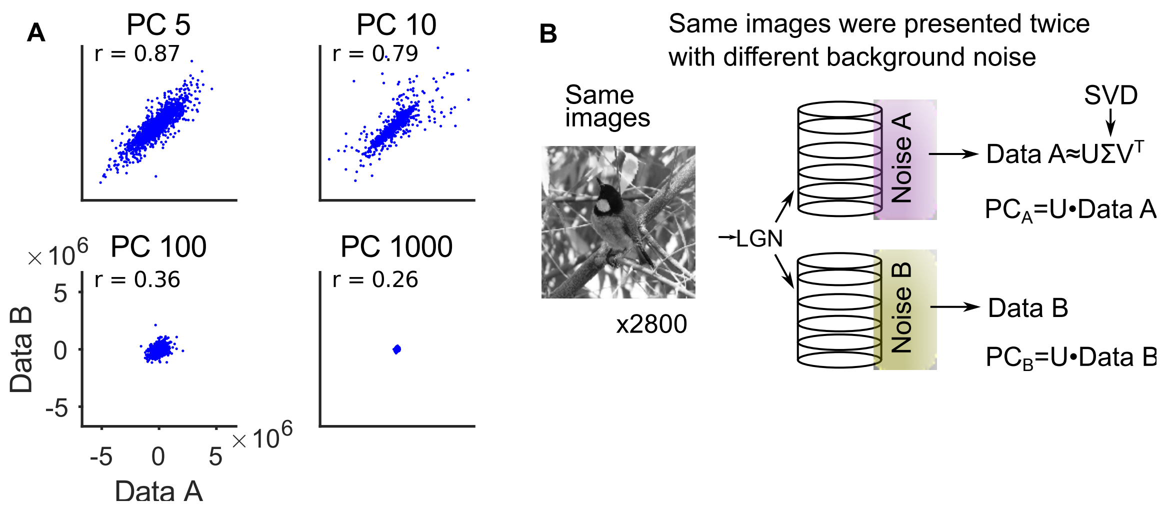

Figure S2: Schematic diagram of cvPCA (cross-validated PCA) to extract signal and noise according to (*11*). (A) Correlations (r) of neural responses for two presentations of the same natural image, using our data-driven noise model with $s = q = 2$ and projected onto selected principal components (PC). **(B)** Schematic diagram of cvPCA (cross-validated PCA). A set of generic images was presented twice with independently drawn noise for each image (Noise A and Noise B). Neural responses to the first presentation (Data A) were factorized by singular value decomposition (SVD) to estimate eigenspectrum of neural responses.

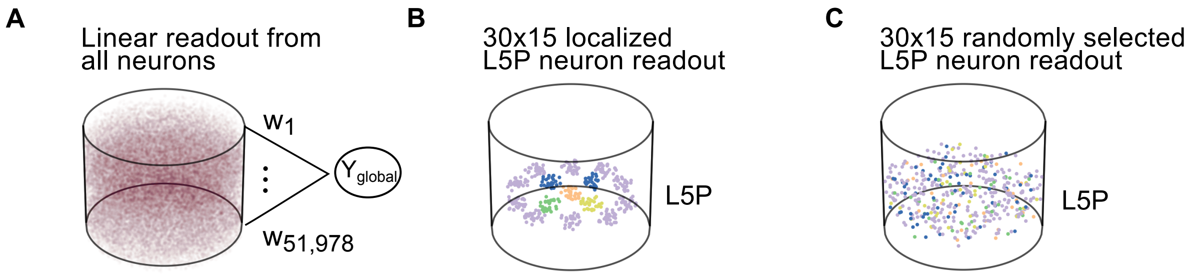

Figure S3: Schematic diagram of different readout scenarios. (A) Schematic diagram of a linear readout receives synaptic input from all 51,978 neurons in the microcircuit model, using a corresponding number of weights that can all be optimized for one particular task. **(B)** 15 spatially separated groups of 30 pyramidal neurons in L5 were selected to signal specific network outputs for 5 different tasks. Color encodes for 5 chosen tasks, same as in Fig. 2A. **(C)** Alternative selection of these 15 populations in L5 without spatial clustering leads to very similar performance (an average accuracy of 94.67% instead of 94.28% for our standard readout convention from **(B)**).

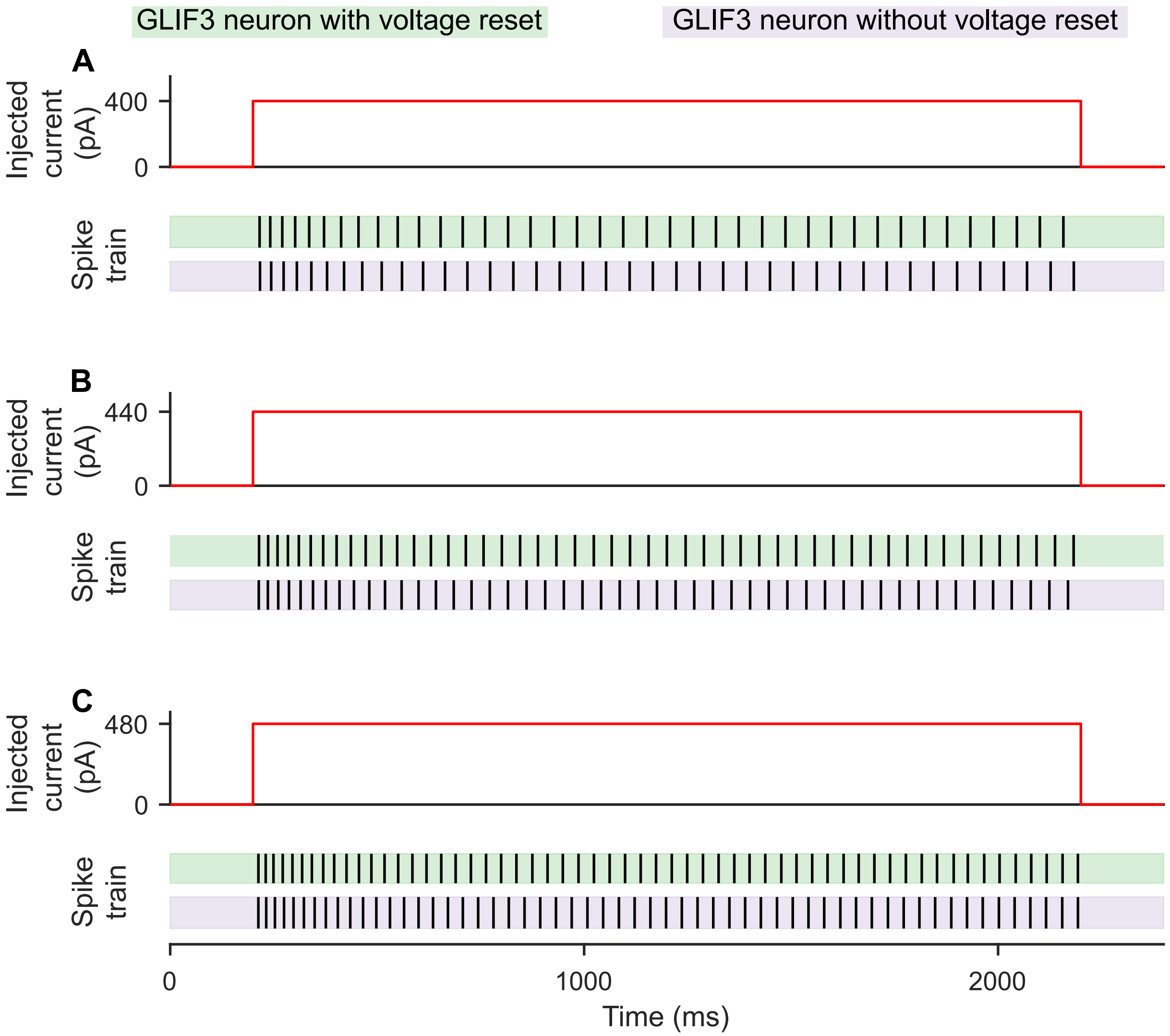

Figure S4: Adding voltage reset does not significantly affect the spike response of the GLIF$_3$ model. (A) Stimulus in form of a step function (top) was inputted to the GLIF$_3$ model with voltage reset used in this study (middle) and the GLIF$_3$ model without voltage reset in (*35*). The after-spike currents in the GLIF3 model cause a spike-frequency adaptation, i.e., they reduce the firing rate during the later part of the input current injection. **(B-C)** Same as in **(A)** but for two different intensities of step stimuli. The difference between two models is so small that it can be ignored. Henceforth, we call our modified GLIF$_3$ model with voltage reset also as GLIF$_3$ model for convenience.

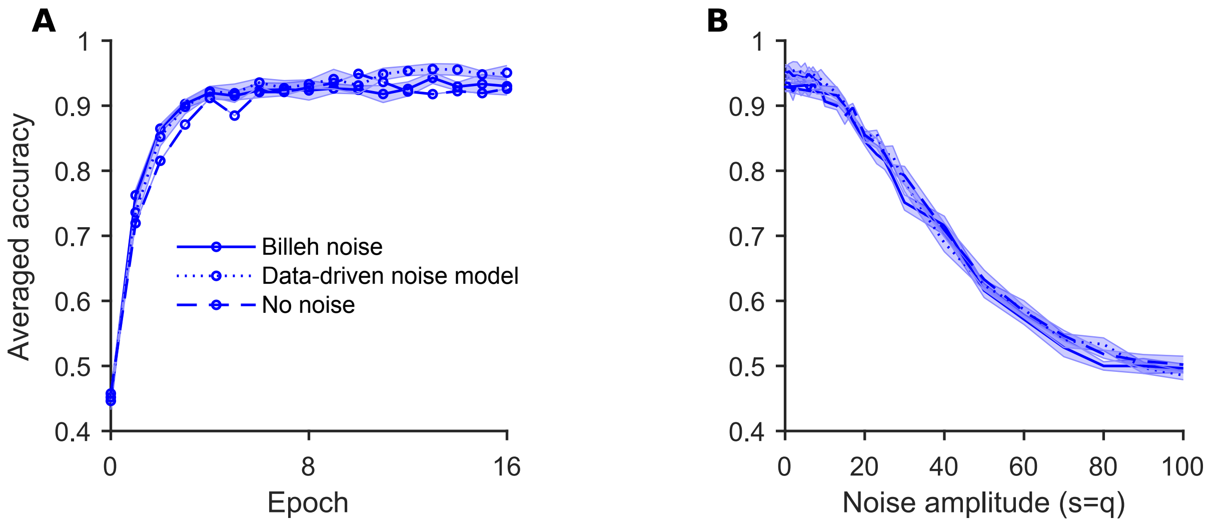

Figure S5: The effect of different noise scenarios during training on testing accuracy. (A) Accuracy on test examples of the V1 model trained with three noise scenarios: i) Single Poisson source, the noise used in (*1*) (Billeh noise), ii) data-driven noise model when $s = q = 2$, iii) no injected noise (No noise). The test accuracy is not sensitive to them. We used the data-driven noise model with $s = q = 2$ during the testing process. **(B)** Decay of accuracy of the V1 model trained with three different noise scenarios for increasing amplitudes of internal noise, see Fig. 3E for a preceding analysis for the V1 model. One sees that these noise scenarios do not dramatically affect the noise robustness of the V1 model.

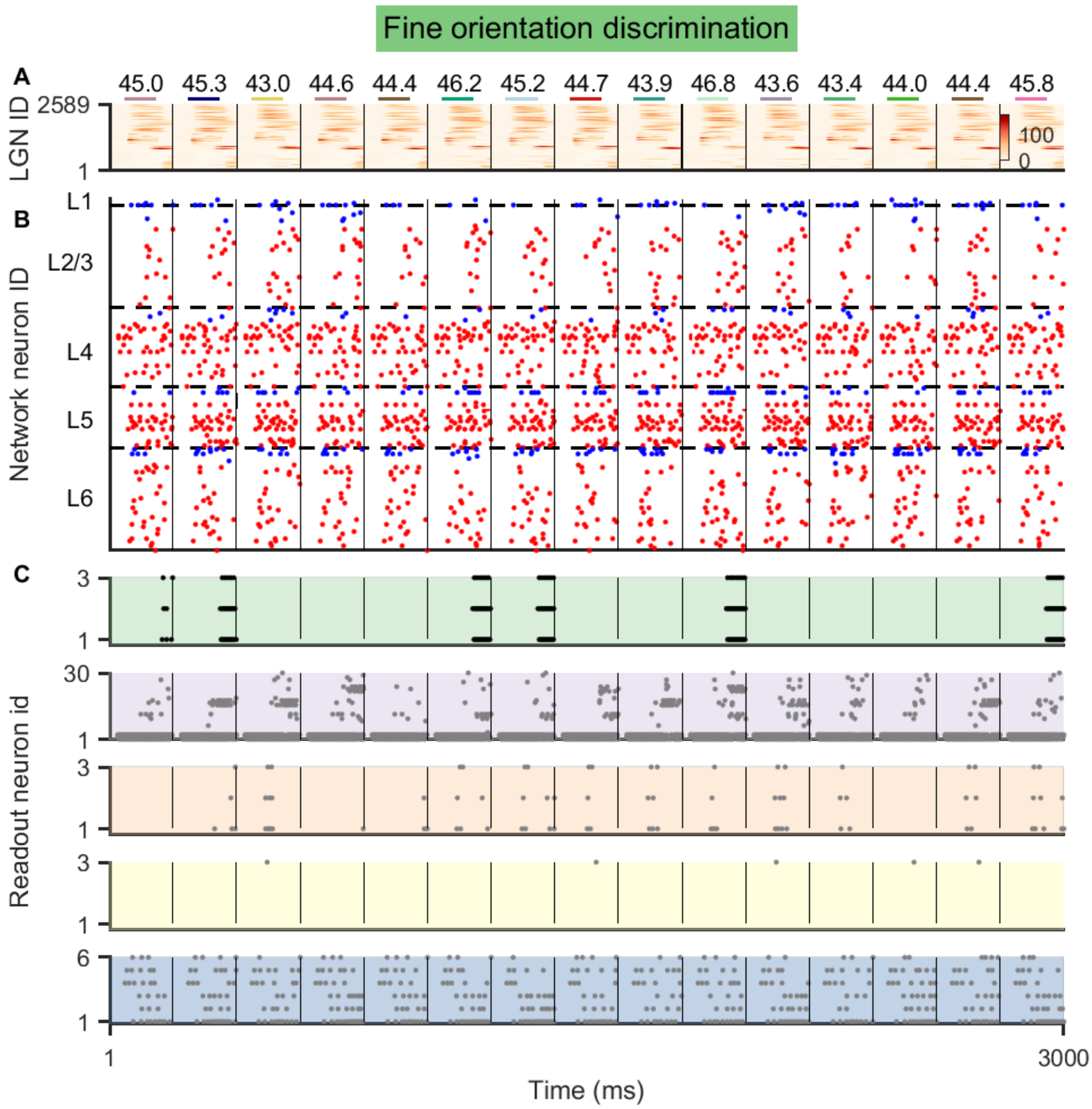

Figure S6: Sample run of the V1 model on input for the fine orientation discrimination task. (A) Colored lines represent the timing of input images. Numbers on them represent the orientations of input gratings. The bottom colormap demonstrates the activity of LGN neuron activity. **(B)** Spike raster of the laminar V1 model. 200 neurons are sampled. Red and blue dots represent the spikes of excitatory and inhibitory neurons, respectively. Note that the spike and membrane potential of the model was reset to 0 after one classification was done (separated by the thick black line). **(C)** Spike raster of readout neurons. 10% of neurons are sampled in every readout population. Color codes of panels are the same as in Fig. 2A. From the top to bottom, there are readout populations of the fine orientation discrimination, the image classification, the visual change detection of nature images and gratings, and the evidence accumulation tasks.

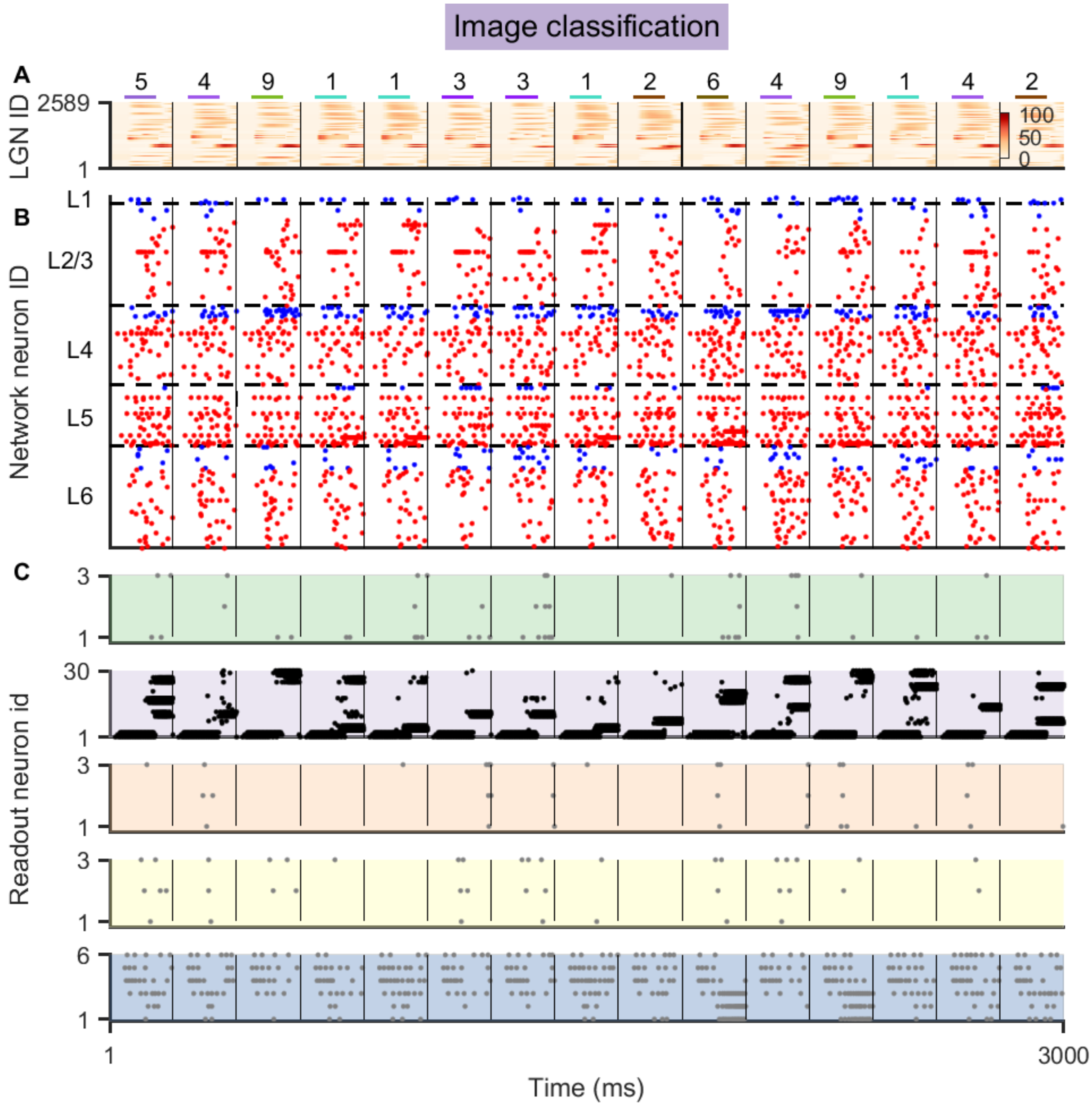

Figure S7: Sample run of the V1 model on input for the image classification task. (A) Colored lines represent the timing of input images. Numbers on them represent the digits in the input images. The bottom colormap demonstrates the activity of LGN neuron activity. **(B)** Spike raster of the laminar V1 model. 200 neurons are sampled. Red and blue dots represent the spikes of excitatory and inhibitory neurons, respectively. Red and blue dots represent the spikes of excitatory and inhibitory neurons, respectively. Note that the spike and membrane potential of the model was reset to 0 after one classification was done (separated by the thick black line). **(C)** Spike raster of readout neurons. 10% of neurons are sampled in every readout population. Color codes of panels are the same as in Fig. 2A. From the top to bottom, there are readout populations of the fine orientation discrimination, the image classification, the visual change detection of nature images and gratings, and the evidence accumulation tasks.

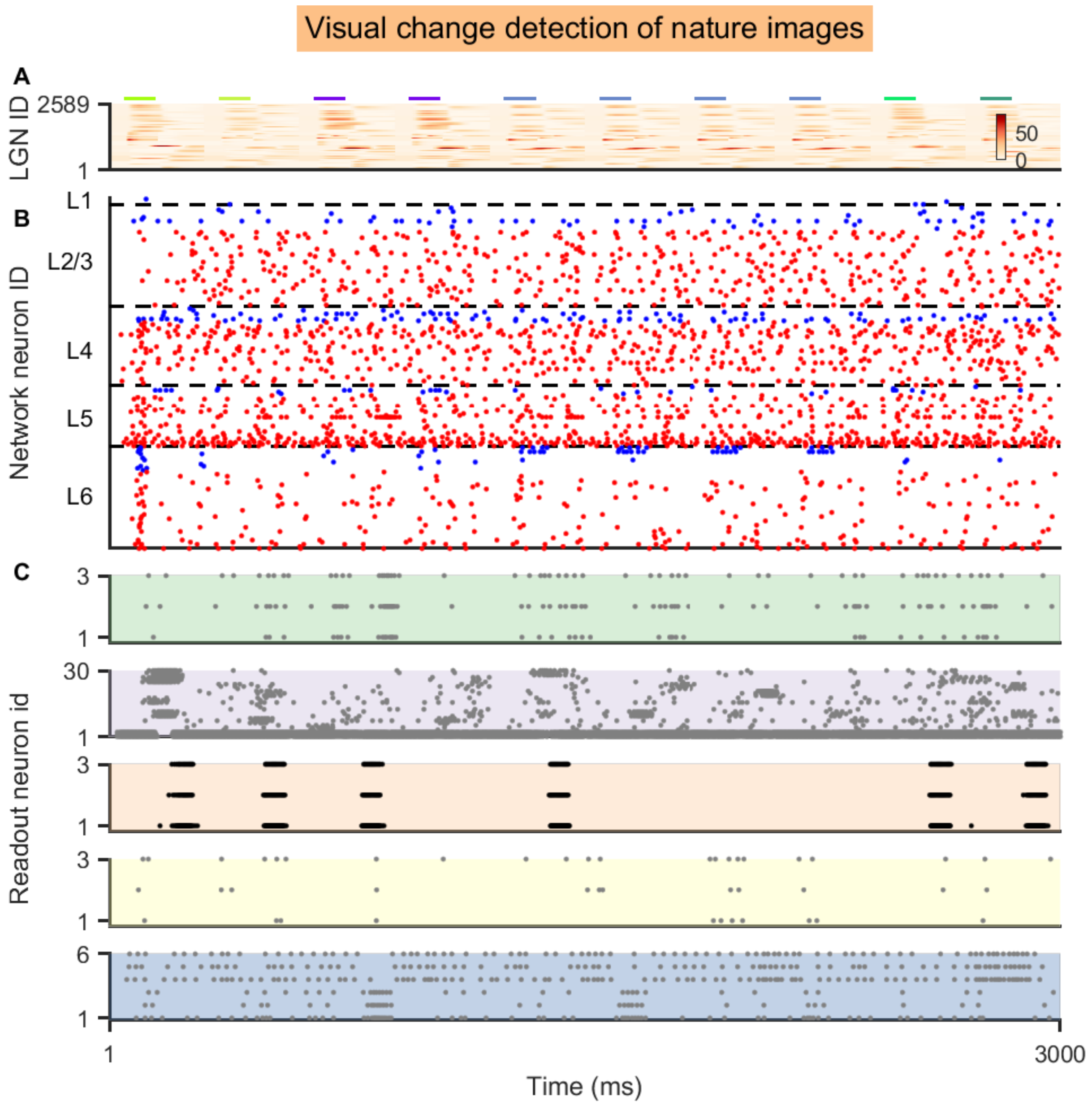

Figure S8: Sample run of the V1 model on input for the visual change detection task of natural images. (A) Colored lines represent the timing of input images and the colors code the image identity. The bottom colormap demonstrates the activity of LGN neuron activity. **(B)** Spike raster of the laminar V1 model. 200 neurons are sampled. The slow noise was resampled every 600 ms. Red and blue dots represent the spikes of excitatory and inhibitory neurons, respectively. **(C)** Spike raster of readout neurons. 10% of neurons are sampled in every readout population. Color codes of panels are the same as in Fig. 2A. From the top to bottom, there are readout populations of the fine orientation discrimination, the image classification, the visual change detection of nature images and gratings, and the evidence accumulation tasks.

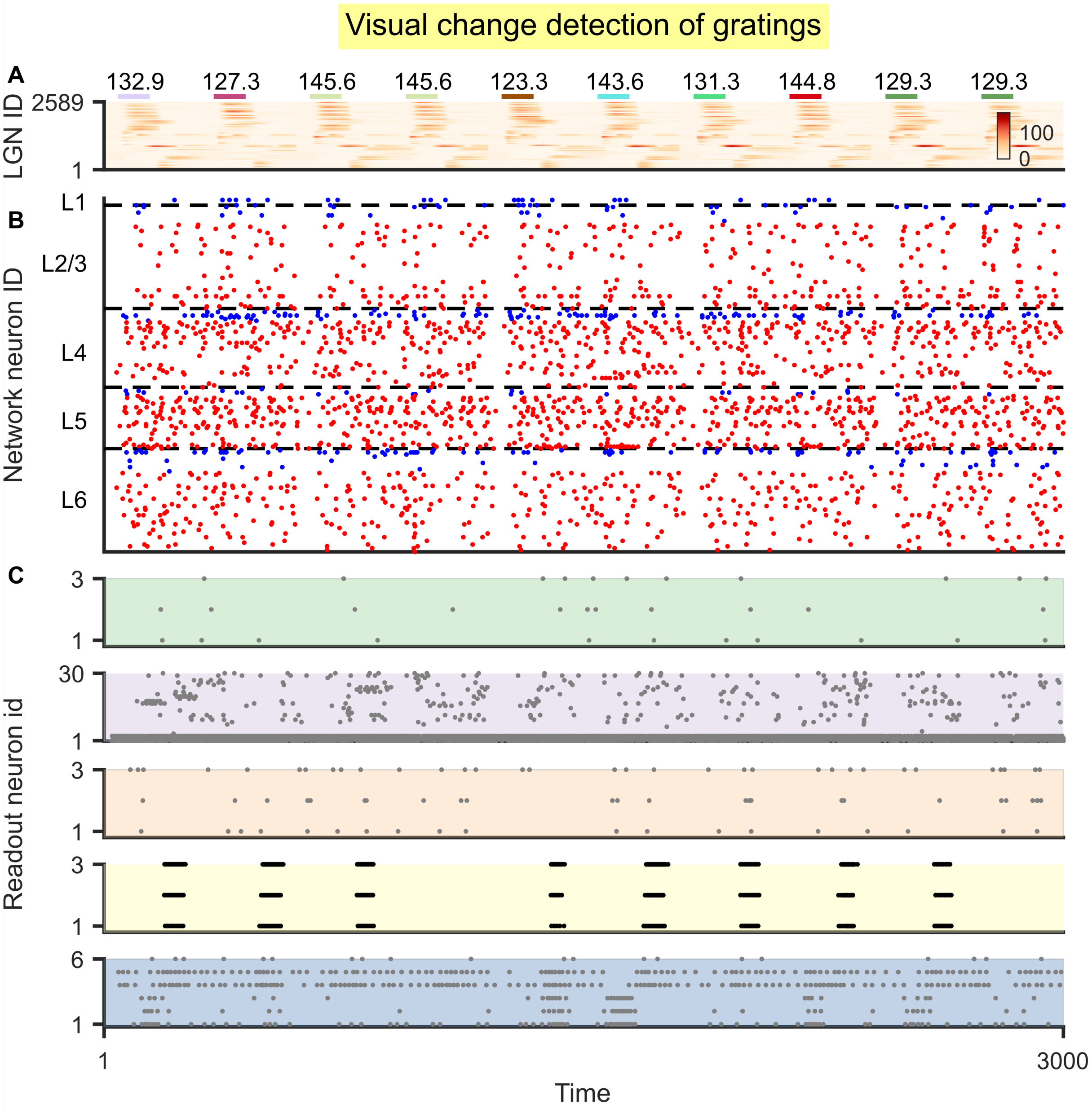

Figure S9: Sample run of the V1 model on input for the visual change detection task of gratings. **(A)** Colored lines represent the timing of input images and the colors code the image identity. Numbers on them represent the orientations of input gratings. The bottom colormap demonstrates the activity of LGN neuron activity. **(B)** Spike raster of the laminar V1 model. 200 neurons are sampled. The slow noise was resampled every 600 ms. Red and blue dots represent the spikes of excitatory and inhibitory neurons, respectively. **(C)** Spike raster of readout neurons. 10% of neurons are sampled in every readout population. Color codes of panels are the same as in Fig. 2A. From the top to bottom, there are readout populations of the fine orientation discrimination, the image classification, the visual change detection of nature images and gratings, and the evidence accumulation tasks.

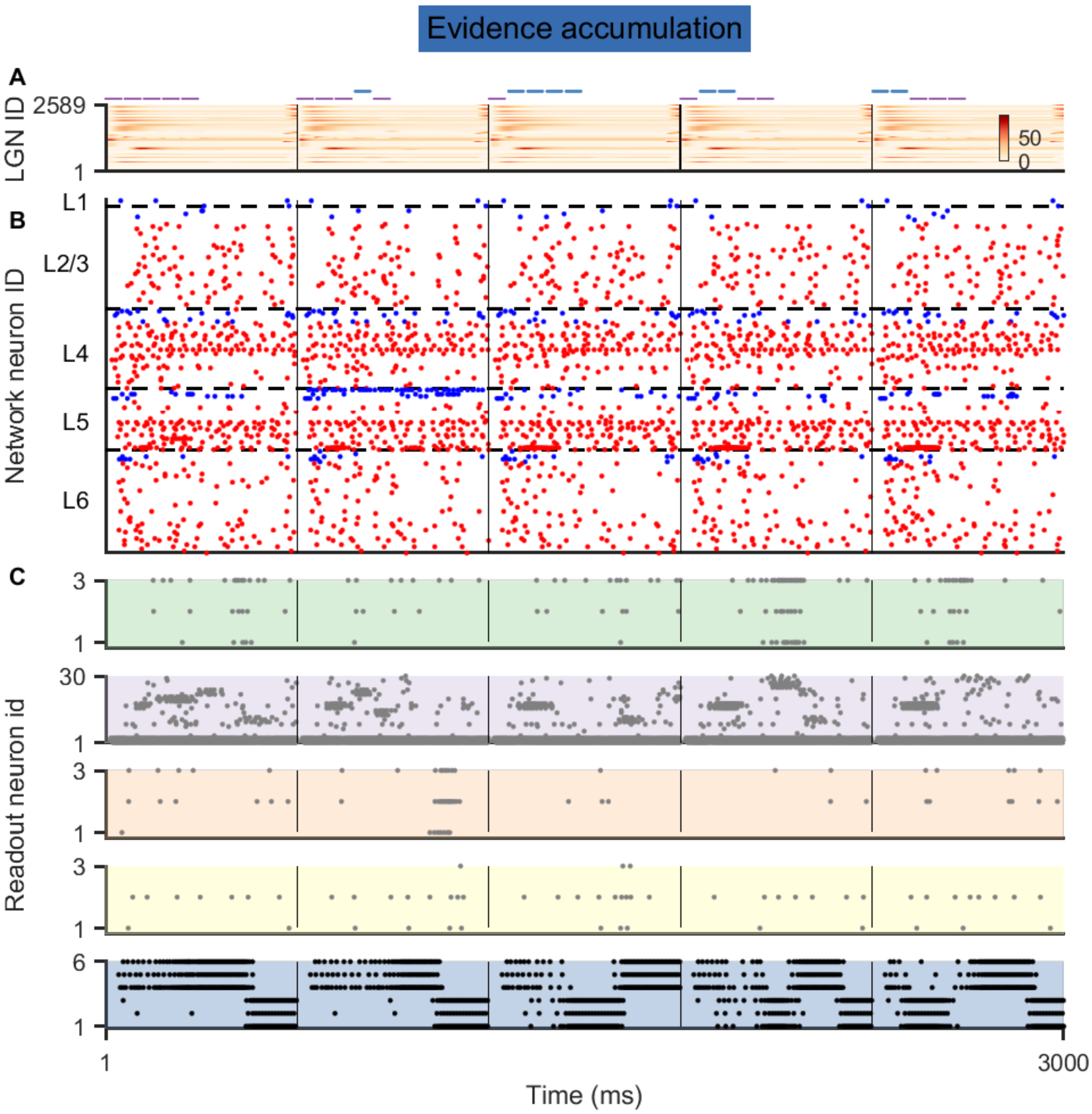

Figure S10: Sample run of the V1 model on input for the evidence accumulation task. (A) Colored lines represent the timing of input left/right cues. The bottom colormap demonstrates the activity of LGN neuron activity. **(B)** Spike raster of the laminar V1 model. Red and blue dots represent the spikes of excitatory and inhibitory neurons, respectively. Note that the spike and membrane potential of the model was reset to 0 after one classification was done (separated by the thick black line). **(C)** Spike raster of readout neurons. 10% of neurons are sampled in every readout population. Color codes of panels are the same as in Fig. 2A. From the top to bottom, there are readout populations of the fine orientation discrimination, the image classification, the visual change detection of nature images and gratings, and the evidence accumulation tasks.

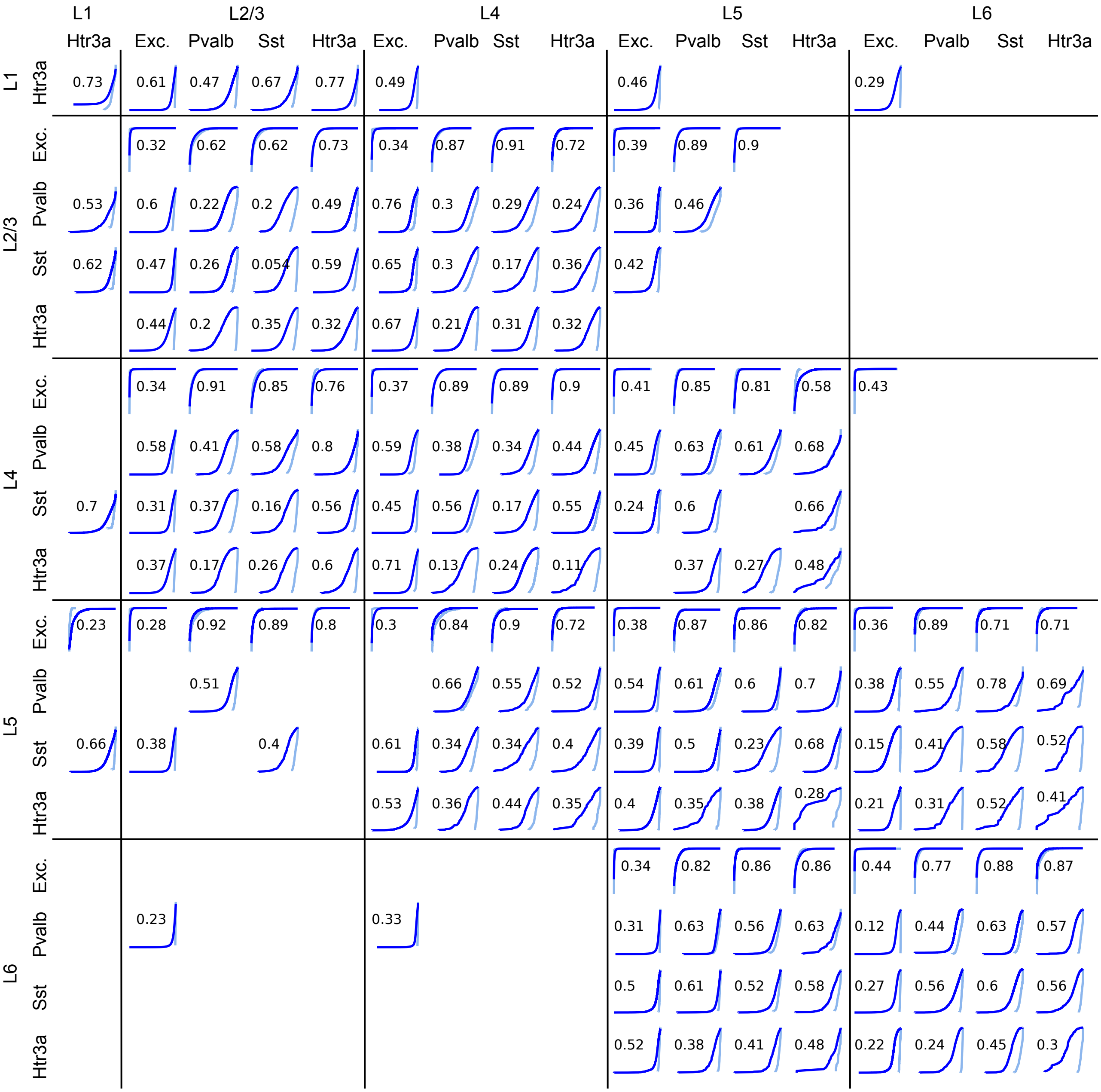

Figure S11: Distribution of recurrent weights between each pair of populations before (light blue) and after learning (dark blue) the 5 tasks. Each row represents a pre-synaptic neuron population, and each column represents a post-synaptic neuron population. The histogram represents the distribution of synaptic weights of all synaptic connections that share the same pre-synaptic and post-synaptic neuron population. Vertical axis in each panel is log-scale. Horizontal axis is linear scale and horizontal range is from the smallest value to the largest value of each population. The number is $1 - D$ where D is from the Kolmogorov–Smirnov test, quantifying the similarity between distributions (*1*). Exc., excitatory neurons.

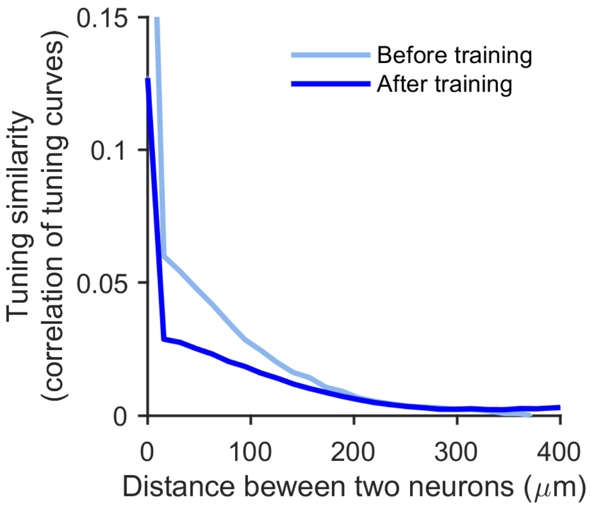

Figure S12: Spatial clustering of orientation tuning of excitatory neurons in L2/3 of the V1 model before and after training. The correlation of tuning curves of two neurons as a function of the horizontal distance between them. Firstly, we calculated the spike counts of L2/3 excitatory neurons in 100 ms when the model received gratings with different orientation (0 to 180° with the step of 5°). Then we calculated the correlation between the tuning curves of neuron pairs and plotted the correlation coefficient as the function of the horizontal distance between the neuron pairs. The spatial cluster is weaker than the experimental results in Fig. 2b of (*22*), probably because they calculated the correlation based on the joint tuning of neurons in the orientation and spatial frequency plane but we did not use the spatial frequency (we do not have a related task).

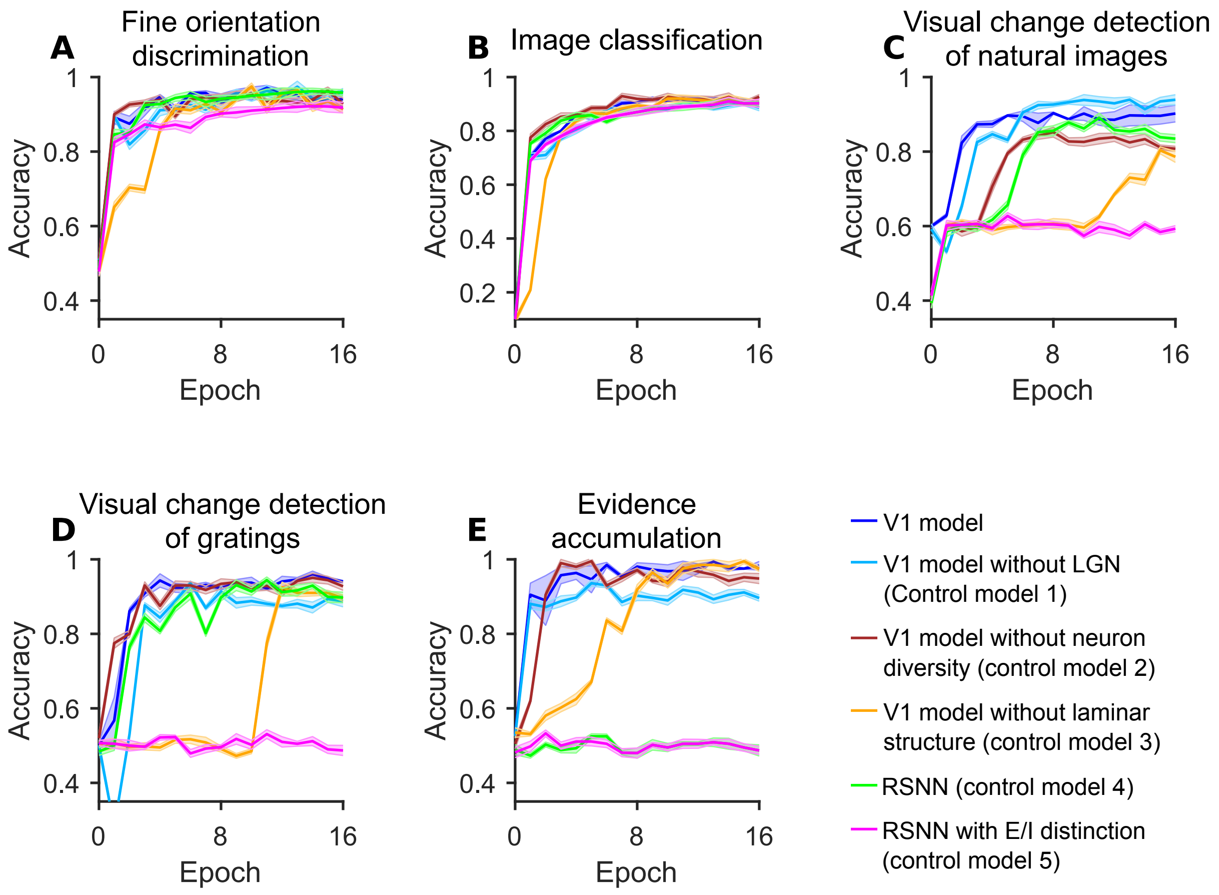

Figure S13: Task-wise accuracy on test examples for the V1 model and 5 control models, all trained for all 5 tasks. (A-E) The testing accuracy is shown for each task as function of training length (number of epochs). Shaded areas represent the SEM over 5 runs.

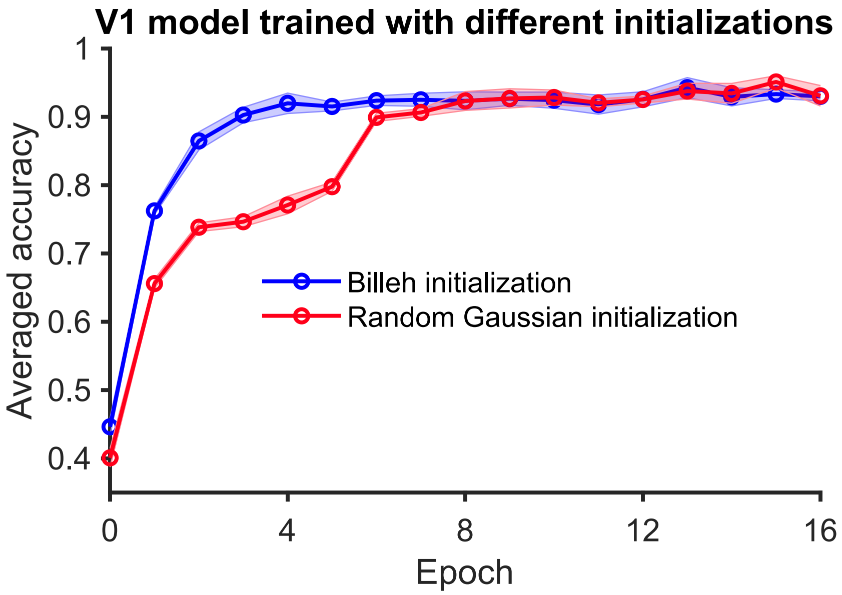

Figure S14: The effect of initialization of synaptic weights on the testing accuracy. The accuracy of V1 model trained with initialization of synaptic weights from (*1*) (Billeh initialization) and random Gaussian initialization whose mean and SD are equal to the Billeh initialization.

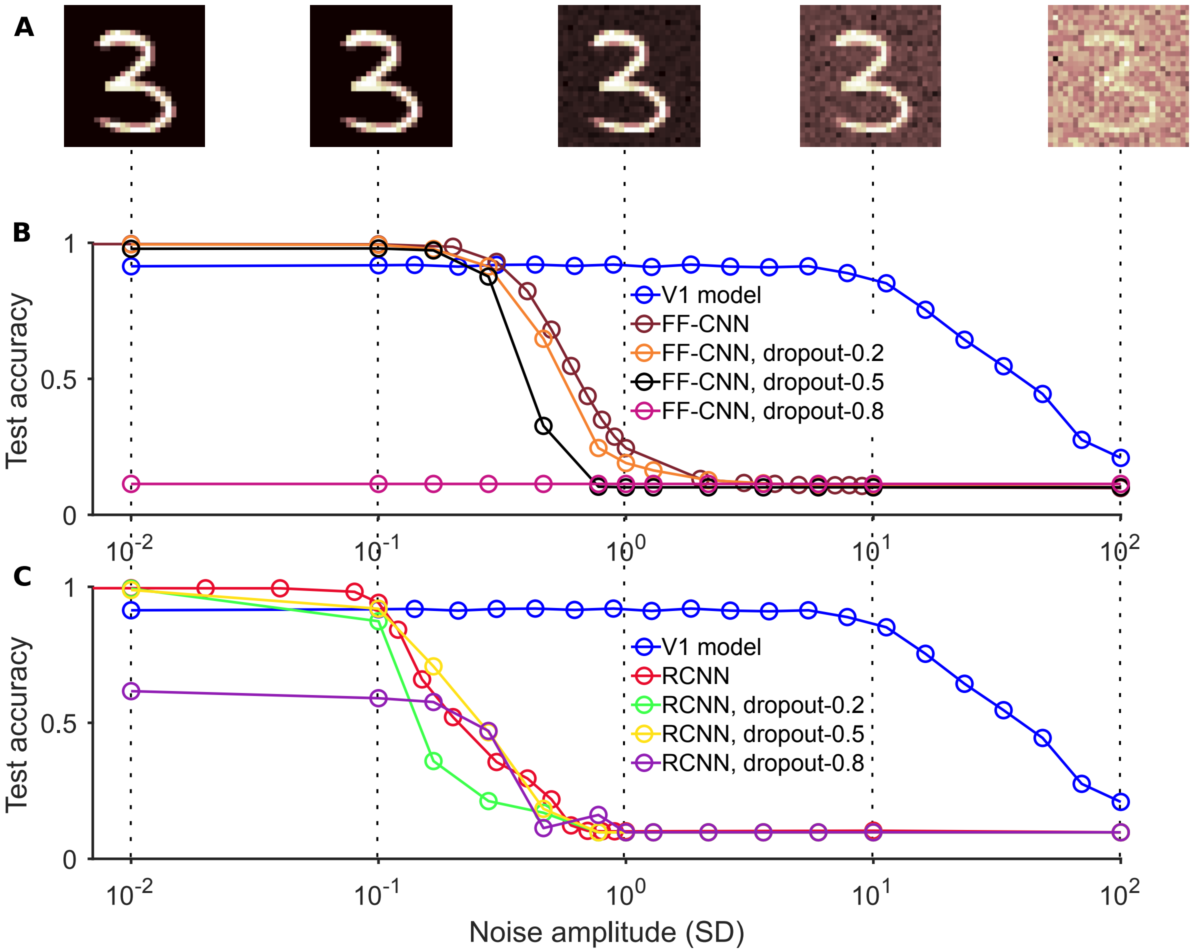

Figure S15: Robustness of CNNs trained with dropout to noise in test images. **(A)** Samples of handwritten MNIST digits with Gaussian noise drawn independently from $\mathcal{N}(0, \mathrm{SD})$ for each pixel, for different values of the SD. **(B)** To add internal noise during training of CNNs, we randomly set some of the elements of the input tensor with probability p using samples from a Bernoulli distribution. Each element was set as zero independently. We use dropout-p to represent this module in the legend. With dropout, the performance of feedforward (FF)-CNNs is still less robust than the V1 model when p is from 0.1 to 0.9. **(C)** Similar to **(B)** but for recurrent CNN (RCNN).

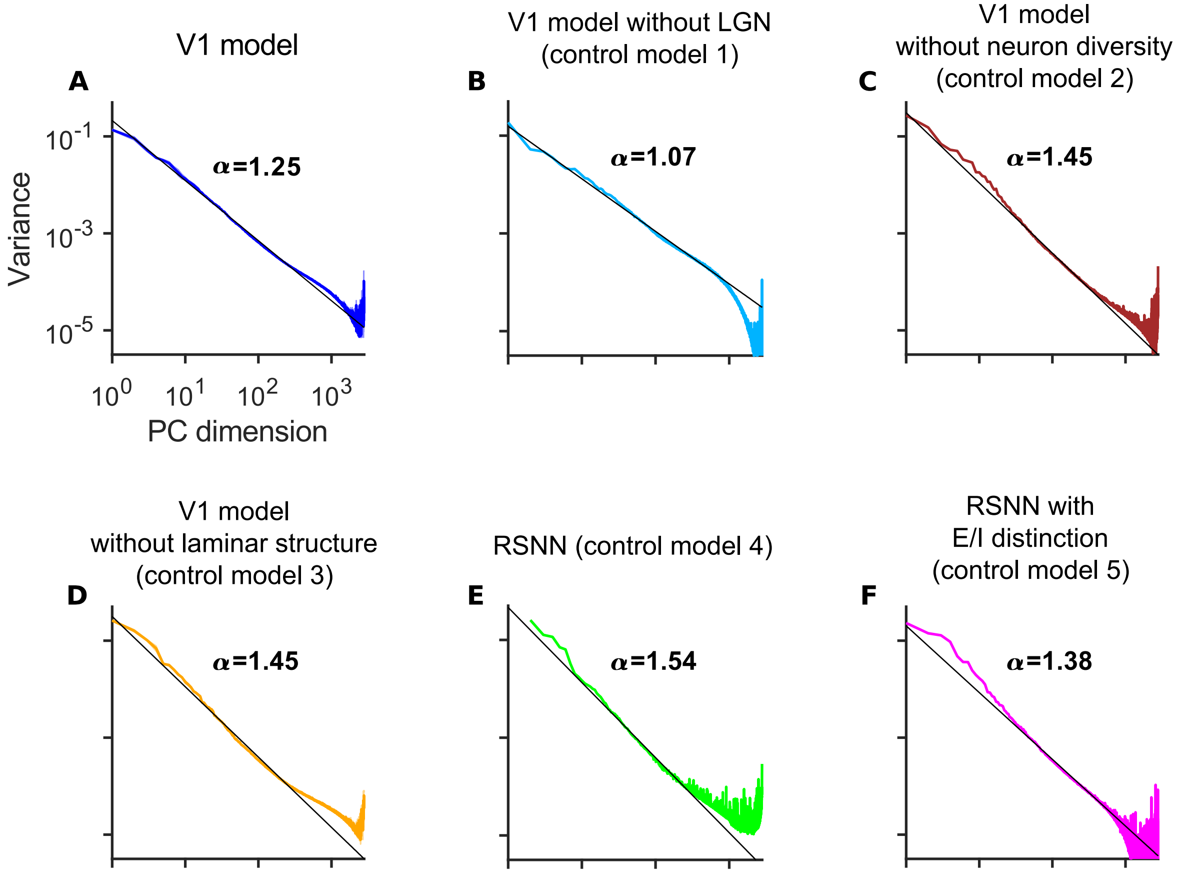

Figure S16: Comparing the eigenspectra of neural codes in the V1 model and control models. (A-F) Eigenspectra of V1 model and 5 control models. In control models except the one without LGN (control model 1), their power-law fittings are worse than that of V1 model. Either head or tail cannot be fitted, making the power-law span about one order of magnitude smaller than that of V1 model. Moreover, the fitted power-law exponent is more away from the experimental data $(1 + 2/d)$.

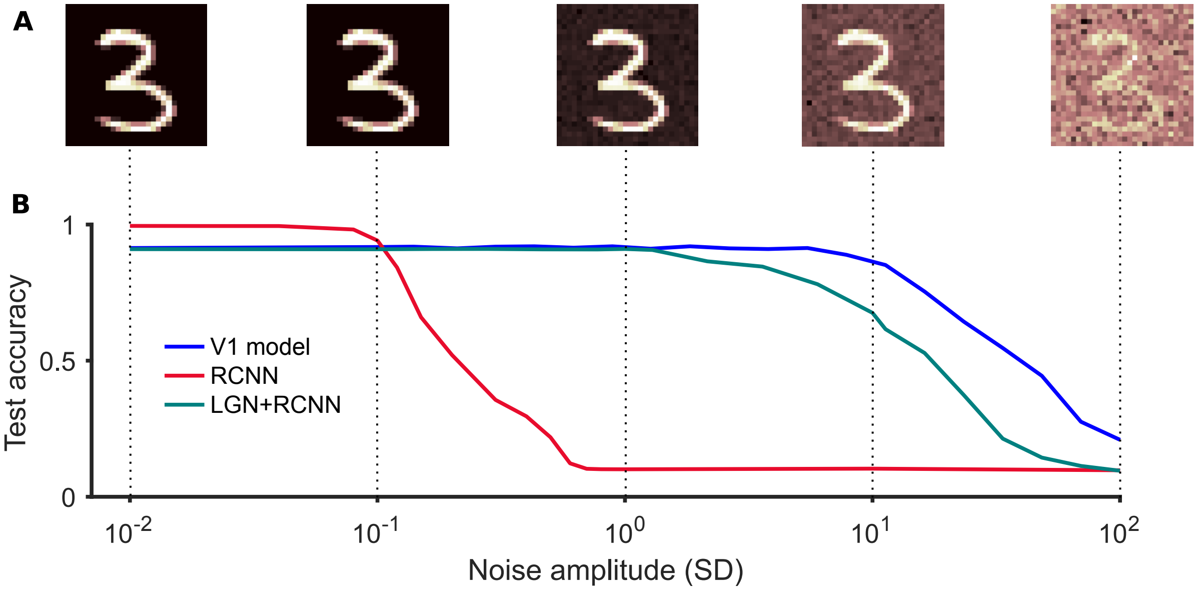

Figure S17: Robustness of CNNs with LGN to noise in images. **(A)** Samples of handwritten MNIST digits with Gaussian noise drawn independently from $\mathcal{N}(0, \mathrm{SD})$ for each pixel, for different values of the SD. **(B)** Adding the LGN model as preprocessor of the RCNN substantially improved their robustness, although it slightly reduces accuracy with little or no noise.

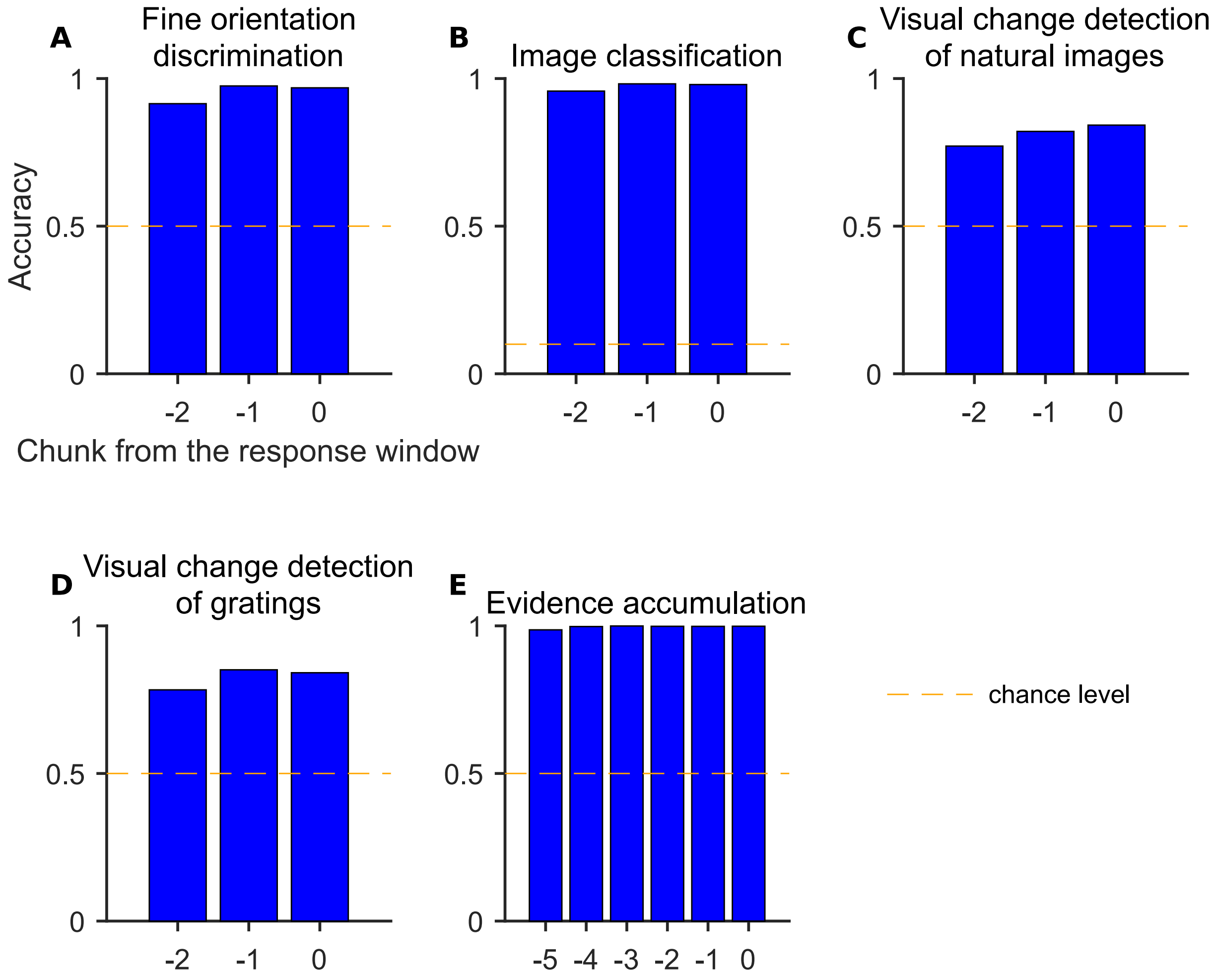

Figure S18: Excitatory neurons in L2/3 contain and transmit most of the information that is needed to solve the 5 computational tasks. To demonstrate that, we trained a multi-layer perceptron (which might be seen as proxy for higher cortical areas that are not represented in the model) that receives the spike counts during a time window of 50 ms from all excitatory neurons in L2/3 of the trained V1 model. We label the response window for each task as 0, and also show results for 50 ms time windows before that (counting backward). The weights of the multi-layer perceptron were trained by Adam optimizer to produce the target output for each task. The multi-layer perceptron has 3 layers, with 3000 and 600 ReLu neurons on the first 2 layers, and the number of neurons on the last layer equal to the number of possible decisions for the 5 tasks. The number of training epochs is 6; the learning rate is 0.0001. **(A-E)** The readout network can transform the firing activity of L2/3E neurons into the target outputs for all 5 tasks with high accuracy. This proves that the firing activity of the L2/3E neurons contains most of the information needed for solving the 5 tasks.

Supplementary Note 1

The differences of readout scenarios can explain why behavioral performance lags behind neural coding fidelity in area V1. The behavioral discrimination threshold for orientations in the mouse V1 was according to (*4*) almost 100 times larger than the discrimination threshold which they inferred from neural coding fidelity of populations of 50,000 neurons in area V1 of the mouse. They conjectured that this difference was caused by the limitations of downstream decoders. The V1 model suggests a further factor that is relevant. Direct measurements of coding fidelity based on simultaneous recordings from 50,000 neurons do not account for the fact that their information content has to be extracted by neurons in V1 that project to downstream areas. These simultaneous recordings are conceptually related to the postulate of having a global readout that receives synaptic input from all 50,000 neurons, see Fig. S3A. However, we have demonstrated in the V1 model that a global linear readout attains for the fine orientation discrimination task an accuracy of 98.18% (the global linear readout layer is trained for 6 epochs with the frozen V1 model whose synaptic weights were trained in our default manner, simulating that the readout from the recorded neurons). On the other hand, a pool of 30 projection neurons on L5 could only achieve an accuracy of 93.17% if one assumed that they were localized closely together (Fig. S3B), and of 94.01% if they were assumed to be randomly distributed in L5 (Fig. S3C). These results suggest that how information from area V1 is extracted and projected to downstream areas is also a limiting factor that is likely to contribute to the gap between the performance of an ideal observer of neural activity in V1 and the behavioral performance of mice.
